# Lipoprotein(a) induces caspase-1 activation and IL-1 signaling in human macrophages

**DOI:** 10.3389/fcvm.2023.1130162

**Published:** 2023-05-24

**Authors:** Martina B. Lorey, Amer Youssef, Lauri Äikäs, Matthew Borrelli, Martin Hermansson, Julia M. Assini, Aapeli Kemppainen, Hanna Ruhanen, Maija Ruuth, Sampsa Matikainen, Petri T. Kovanen, Reijo Käkelä, Michael B. Boffa, Marlys L. Koschinsky, Katariina Öörni

**Affiliations:** ^1^ Atherosclerosis Research Laboratory, Wihuri Research Institute, Helsinki, Finland; ^2^ Robarts Research Institute, Schulich School of Medicine & Dentistry, The University of Western Ontario, London, ON, Canada; ^3^ Department of Physiology & Pharmacology, Schulich School of Medicine & Dentistry, The University of Western Ontario, London, ON, Canada; ^4^ Molecular and Integrative Biosciences, Faculty of Biological and Environmental Sciences, University of Helsinki, Helsinki, Finland; ^5^ Helsinki University Lipidomics Unit (HiLIPID), Helsinki Institute of Life Science (HiLIFE) and Biocenter Finland, Helsinki, Finland; ^6^ Helsinki Rheumatic Disease and Inflammation Research Group, University of Helsinki and Helsinki University Hospital, Helsinki, Finland; ^7^ Department of Biochemistry, Schulich School of Medicine & Dentistry, The University of Western Ontario, London, ON, Canada

**Keywords:** inflammation, lipoprotein(a), apolipoproteins, atherosclerotic cardiovascular disease, atherosclerosis, caspase-1, interleukin-1 family

## Abstract

**Introduction:**

Lipoprotein(a) (Lp(a)) is an LDL-like particle with an additional apolipoprotein (apo)(a) covalently attached. Elevated levels of circulating Lp(a) are a risk factor for atherosclerosis. A proinflammatory role for Lp(a) has been proposed, but its molecular details are incompletely defined.

**Methods and results:**

To explore the effect of Lp(a) on human macrophages we performed RNA sequencing on THP-1 macrophages treated with Lp(a) or recombinant apo(a), which showed that especially Lp(a) induces potent inflammatory responses. Thus, we stimulated THP-1 macrophages with serum containing various Lp(a) levels to investigate their correlations with cytokines highlighted by the RNAseq, showing significant correlations with caspase-1 activity and secretion of IL-1β and IL-18. We further isolated both Lp(a) and LDL particles from three donors and then compared their atheroinflammatory potentials together with recombinant apo(a) in primary and THP-1 derived macrophages. Compared with LDL, Lp(a) induced a robust and dose-dependent caspase-1 activation and release of IL-1β and IL-18 in both macrophage types. Recombinant apo(a) strongly induced caspase-1 activation and IL-1β release in THP-1 macrophages but yielded weak responses in primary macrophages. Structural analysis of these particles revealed that the Lp(a) proteome was enriched in proteins associated with complement activation and coagulation, and its lipidome was relatively deficient in polyunsaturated fatty acids and had a high n-6/n-3 ratio promoting inflammation.

**Discussion:**

Our data show that Lp(a) particles induce the expression of inflammatory genes, and Lp(a) and to a lesser extent apo(a) induce caspase-1 activation and IL-1 signaling. Major differences in the molecular profiles between Lp(a) and LDL contribute to Lp(a) being more atheroinflammatory.

## 1. Introduction

Atherosclerotic cardiovascular diseases (ASCVDs) are the major causes of death in the Western and westernized world and are caused by the accumulation of plasma lipoproteins in the arterial wall (1). Circulating plasma lipoproteins are spherical particles which contain a hydrophobic core and an amphiphilic surface. The core contains cholesteryl esters (CE) and triacylglycerols (TAG), while the surface is composed of a monolayer of phospholipids, in which apolipoproteins are embedded. Such molecular particle architecture allows the hydrophobic lipids to be carried in the bloodstream as isolated packages in a metabolically regulated way. Lipoproteins containing apolipoprotein (apo) B are mainly low-density lipoprotein (LDL) particles. Increased plasma levels of the particles lead to their increased entry into the intimal areas of atherosclerosis-prone arterial segments. The intimal accumulation of cholesterol and the ensuing local inflammatory reaction leads to the formation of atherosclerotic lesions, which ultimately may be transformed into vulnerable plaques susceptible to rupture and atherothrombotic clinical complications (2, 3).

Lipoprotein (a) (Lp(a)) is an unconventional cholesterol-rich lipoprotein that resembles LDL, but where the apoB is covalently bound to apo(a) (4). It was first described as a polymorphic form of LDL (5), but since then it has become evident that elevated levels of Lp(a) (>50 mg/dl) are an independent and causal risk factor for ASCVD (6–8). The prevalence of Lp(a) levels >50 mg/dl varies amongst populations but is overall estimated to be 20% globally (9). The details of the biosynthesis, function, and catabolism of this particle are not fully understood. What is known is that Lp(a) promotes inflammation in the vessel wall in various ways (10, 11). It induces endothelial dysfunction through apo(a) by increasing endothelial cell contraction and permeability (12), and by stimulating the expression of pro-inflammatory factors (13). Potent reduction of Lp(a) has been shown to alter the inflammatory signatures of circulating monocytes at the transcriptome level (14). Macrophages play a crucial role in different stages of atherogenesis (15–18), and they are the most populous group of cells in atherosclerotic plaques (19). Lp(a) activates macrophages and promotes the pro-inﬂammatory, M1-type phenotype which in turn secretes interleukin-8 (IL-8) (20, 21) and induces apoptosis in ER-stressed macrophages in advanced atheromas (22).

There is a body of evidence suggesting that oxidized phospholipids (OxPLs) that are preferentially carried by Lp(a) signiﬁcantly contribute to, or even primarily account for, the atherogenicity of the particle and the increased risk it confers for ASCVD (23). OxPLs are able to boost inflammation by inducing a “hypermetabolic” state in phagocytes that amplifies the production of IL-1β (24). It has been estimated that the covalently- and noncovalently-associated oxPLs associated with Lp(a) are present in roughly equal proportions and together represent approximately 85% of the oxPL carried in plasma (25). One OxPL, 1-palmitoyl-2-(5-oxovaleroyl)-sn-glycero-3-phosphocholine (POVPC) has been shown to upregulate cytokine genes, when added in amounts of 10 µM to macrophages (26).

In the present study, we evaluated changes in the transcriptome of THP-1 macrophages induced by Lp(a) and recombinant apo(a). We then analyzed correlations between serum Lp(a) levels with the secretion of a subset of IL-10-related cytokines by incubating human serum samples containing various Lp(a) levels with THP-1 macrophages. We further isolated Lp(a) and LDL from three volunteer donors with elevated circulating levels of Lp(a) and analyzed their molecular composition and pro-inflammatory potential when added to cultured primary or THP-1-derived macrophages. Our multifaceted approach allowed us to uncover differences between Lp(a) and LDL in a setting of an identical genetic and lifestyle background.

## 2. Materials and methods

### 2.1. Lp(a) and apo(a) isoform determination

Fasting blood samples were obtained from subjects who provided written informed consent. The study was approved by the Research Ethics Boards of the University of Western Ontario and the Medical Ethical Committee of the Helsinki and Uusimaa hospital district.

Plasma levels of total cholesterol, HDL-cholesterol, and TAG were determined by enzymatic analyses (Indiko and Konelab System Cholesterol Reagents, Thermo Scientific) and LDL-cholesterol was calculated by the Friedewald formula. ApoB and apoA-I were determined by nuclear magnetic resonance spectroscopy from fresh frozen EDTA blood samples (Nightingale Health Plc, Helsinki, Finland) as previously described (27). Plasma Lp(a) levels were determined using an ELISA kit (Mercodia, 10-1106-01). Apo(a) isoforms were determined by SDS-agarose gel electrophoresis followed by western blot analysis as previously described (28) with the following modifications: 10 µl of undiluted plasma was loaded in each lane; the blots were calibrated using purified recombinant apo(a) variants ranging from 12 to 33 KIV repeats (29); the primary antibody was a mouse monoclonal antibody raised in-house against 17 K recombinant apo(a) and recognizing KIV type 2; the secondary antibody was a sheep polyclonal anti-mouse IgG conjugated to horseradish peroxidase (Cytiva); and immunoreactive bands were visualized by enhanced chemiluminescence (SuperSignal West Femto, Pierce Biochemicals) on a BioRad ChemiDoc Plus imager. For the three, whose LDL and Lp(a) particles were isolated, the apo(a) structure was determined: donor A had the 16-kringle apo(a) isoform with no second isoform detected; donor B had the 17-kringle and 27-kringle apo(a) species, each at similar abundance; and donor C had a single isoform of 17-kringle species.

### 2.2. Protein/lipoprotein isolation and verification

Recombinant 17-kringle apo(a) was expressed in HEK293 cells (RRID:CVCL_0045) and purified from the conditioned medium from these cells by lysine-Sepharose affinity chromatography as previously described (30, 31). For the isolation of LDL and Lp(a), blood was collected from the antecubital vein of healthy volunteers into BD Vacutainer blood collection tubes containing sodium polyanethol sulfonate and acid citrate dextrose. Plasma was obtained through centrifugation of whole blood at 3,000×*g* for 15 min at 4°C. Phenylmethylsufonyl fluoride (PMSF) was added to a final concentration of 0.1 mM and the plasma was rotated at 4°C for 15 min. The plasma was adjusted to a density of 1.063 g/ml with solid sodium bromide and centrifuged at 45,000×*g* for 24 h at 6°C. The top fraction was removed and the infranatant was adjusted to a density of 1.21 g/ml with solid sodium bromide. The sample was centrifuged at 45,000×*g* for 24 h at 6°C and the top layer was isolated and extensively dialyzed against 20 mM Tris-HCl, pH 7.4, containing 150 mM NaCl, 0.01% NaN3, and 0.01% EDTA (Buffer A). The sample was then loaded onto a DEAE-Sepharose Fast Flow (Pharmacia) ion exchange column (2.5 cm × 12 cm column). Lipoproteins were eluted in a stepwise NaCl concentration gradient (50–300 mM NaCl in 20 mM Tris-HCl, pH 7.4). LDL- and Lp(a)-containing fractions (from 110 mM NaCl and 250 mM NaCl elutions, respectively) were pooled and dialyzed against HEPES-buffered saline (HBS; 20 mM HEPES pH 7.4, 150 mM NaCl). Concentration was determined by bicinchoninic acid assay (Pierce BCA Protein Assay Kit, Thermo Scientific, 23225) using BSA as a standard. The integrity of the purified lipoproteins was assessed by SDS-PAGE under non-reducing and reducing conditions followed by staining with Imperial Stain (Sigma). Importantly, there was no cross-contamination of the Lp(a) and LDL preparations.

### 2.3. Cell culture

THP-1 monocytes (ATCC® TIB­202™, RRID:CVCL_0006) were maintained in RPMI 1640 supplemented with 10% fetal bovine serum, 2 mM Glutamax, 100 U/ml penicillin, 100 µg/ml streptomycin, and 25 mM HEPES at 37°C in 5% CO_2_. To induce differentiation of the monocytes into macrophages, 100 nM phorbol 12-myristate 13-acetate (PMA) was added to the media, and the cells were incubated for 72 h (32).

For primary macrophages, peripheral blood mononuclear cells (PBMCs) from three donors per experiment were isolated from buffy coats and differentiated into macrophages as described previously (33). Buffy coats separated from the blood of healthy human donors were obtained from the Finnish Red Cross Blood Service, Helsinki, Finland (supplied under permit no. 21/2020). In total, 1.5 × 10^6^ monocytes were seeded per well on 24-well plates. The monocytes were cultured in serum-free macrophage media (Macrophage-SFM, Gibco, Thermo Fisher Scientific) supplemented with 10 ng/ml granulocyte-macrophage colony-stimulating factor (GM-CSF) (ImmunoTools) and 50 units/ml penicillin/streptomycin (Lonza, Basel, Switzerland) at 37°C and 5% CO_2_ for 6 days to polarize the monocytes into macrophages of the pro-inflammatory M1-phenotype. On day six, the cells were washed with PBS, supplied with fresh RPMI 1640 medium (Gibco) supplemented with l-glutamate and antibiotics, and primed with 1 µg/ml LPS (Sigma, L3012) for 4 h before stimulation.

### 2.4. RNA sequencing of macrophages stimulated with Lp(a) or apo(a)

THP-1 macrophages were grown in PMA-supplemented medium which was refreshed after 36 h. After a further 36 h, this medium was replaced with serum-free complete medium and incubated for 16 h. Following starvation, the medium was replaced with fresh serum-free medium supplemented with Lp(a), 17 K apo(a), or vehicle for 6 h; we used equimolar concentrations of Lp(a) and 17 K apo(a) (250 nmol/L, or 191 and 62.5 µg/ml protein, respectively). Each of the treatments was performed in the absence or presence of 200 mM ε-aminocaproic acid (to inhibit lysine binding) for a total of six treatment groups, performed in duplicate. This represented a single biological replicate for each treatment. Cells were incubated with the treatments for 6 h, after which total RNA was isolated using the QIAGEN RNeasy Mini Kit according to the manufacturer's protocol. The RNA was submitted to the London Regional Genomics Centre (LRGC) for analysis using the Illumina NextSeq 500 (Illumina Inc.). Total RNA samples were quantified using the Qubit 2.0 Fluorometer (ThermoFisher Scientific). Quality was assessed using the Agilent 2100 Bioanalyzer (Agilent Technologies Inc.) and the RNA 6000 Nano kit (Caliper Life Sciences). Samples were then processed using the Vazyme VAHTS Total RNA-seq (H/M/R) Library Prep Kit for Illumina (Vazyme) which includes ribosomal RNA reduction. Samples were depleted and fragmented prior to cDNA synthesis, then cDNA was indexed, cleaned-up and amplified via polymerase chain reaction. Biological replicate libraries were pooled into one library per treatment. The pooled library size distribution was assessed on an Agilent High Sensitivity DNA Bioanalyzer chip (Agilent Technologies Inc.) and quantified using the Qubit 2.0 Fluorometer. The library was sequenced as a single end run, 1 × 76 bp, using a High Output v2 kit (75 cycles). Fastq data files were downloaded from BaseSpace and analyzed using Partek Flow. After importation, data were aligned to hg19 using STAR 2.5.3a and annotated using RefSeq Transcripts 90. Features with more than five reads were normalized using counts per million (CPM), then the -fold changes and *p*-values were determined using Partek Flow's Gene Specific Analysis (GSA). To visualize physical and functional interactions between the transcripts and to determine enriched pathways within the transcript set we used the STRING (“Search Tool for Retrieval of Interacting Genes/Proteins”) version 11.5 (34, 35) (https://string-db.org/) and only relationships with the highest confidence score (0.9) were considered, unconnected nodes were removed from the network.

### 2.5. Cell stimulations and cytokine assays

Activity of caspase-1 was measured using the Caspase-Glo® 1 Inflammasome Assay (Promega Corporation, WI, USA) following the manufacturer's instructions. In brief, macrophages were incubated with Lp(a), LDL, 10% v/v serum, recombinant apo(a), nigericin, or corresponding volume of PBS for 3 h, then the luminogenic caspase-1 substrate was added to the supernatant, and the luminescence resulting from cleavage by active caspase-1 was recorded. The accuracy of the Glo assay was confirmed using the Human Caspase-1/ICE Quantikine ELISA Kit (R&D systems) in selected samples of all experiments. To measure the cellular release of cytokines by macrophages which had been incubated with the indicated amounts of Lp(a), LDL, serum, recombinant 17 K apo(a), or PBS for 3 h, or nigericin for 1 h, the incubation media were collected, and the concentration of IL-1β was measured by ELISA assay (Human DuoSet ELISA for IL-1β). IL-18 was measured by ELISA (Human Total IL-18 DuoSet ELISA for IL-18) or Luminex, the concentrations for IL-1α, IL-6, IL-8, IL-10, IFN-γ, TNF-α, MIF, and TRAIL were determined by Luminex assay (Bio-Rad Bio-Plex Express) according to the manufacturer's instructions.

For inhibitor studies, the caspase inhibitor Z-YVAD-fmk (R&D Systems, 25 µM) or NOD-, LRR- and pyrin domain-containing protein 3 (NLRP3) inflammasome inhibitor MCC950 (Sigma-Aldrich, 1 µM) were added in serum-free medium to the cells 1 h prior to stimulation.

### 2.6. Protein preparation, LC-MS analysis, proteomic data and bioinformatic analysis

To analyze the protein content of isolated Lp(a) and LDL particles, the samples were prepared for MS analysis using the filter-assisted sample preparation protocol (36). For each of the three technical replicates per sample 300 ng of digested proteins was used in the nano-LC-HD-MSE analysis as previously described (37). Briefly, protein digests were analyzed using a nano-LC-Thermo Q Exactive Plus. The peptides were separated by the Easy-nLC system (Thermo Scientific) equipped with a reverse-phase trapping column Acclaim PepMapTM 100 (C18, 75 µm × 20 mm, 3 µm particles, 100 Å pores; Thermo Scientific), followed by an analytical Acclaim PepMapTM 100 RSLC reversed-phase column (C18, 75 µm × 250 mm, 2 µm particles, 100 Å; Thermo Scientific). The injected peptides were trapped at a flow rate of 2 µl min-1 in 100% of solution A (0.1% formic acid). After trapping, the peptides were separated with a linear gradient of 120 min comprising 96 min from 3% to 30% of solution B (0.1% formic acid/80% acetonitrile), 7 min from 30% to 40% of solution B, and 4 min from 40% to 95% of solution B. Each sample run was followed by two empty runs to reduce the sample carryover from previous runs.

LC-MS acquisition data was done with the mass spectrometer settings as follows: The resolution was set to 140,000 for MS scans, and 17,500 for the MS/MS scans. Full MS was acquired from 350 to 1,400 *m*/*z*, and the 10 most abundant precursor ions were selected for fragmentation with 30 s dynamic exclusion time. Ions with 2+, 3+, and 4 + charges were selected for MS/MS analysis. Secondary ions were isolated with a window of 1.2 *m*/*z*. The MS AGC target was set to 3 × 10^6^ counts, whereas the MS/MS AGC target was set to 1 × 10^5^. Dynamic exclusion was set with a duration of 20 s. The NCE collision energy stepped was set to 28 kJ mol^–1^.

Following LC-MS/MS acquisition, the raw data files of each set were qualitatively analyzed by Proteome Discoverer (PD), version 2.4 (Thermo Scientific, USA). The identification of proteins by PD was performed against the UniProt Human protein database (UniProt release 2020_02 downloaded on May 29, 2020, with 20,365 entries) using the built-in SEQUEST HT engine. The following parameters were used: 10 ppm and 0.02 Da were tolerance values set for MS and MS/MS, respectively. Trypsin was used as the digesting enzyme, and two missed cleavages were allowed. Carbamidomethylation of cysteines was set as a fixed modification, while the oxidation of methionine and deamidation of asparagine and glutamine were set as variable modifications. The false discovery rate was set to less than 0.01 and a peptide minimum length of six amino acids. For relative quantitation, apoB-100 was set to 1 as both Lp(a) and LDL contain exactly one copy. Known contaminants, such as keratins, were removed from the data set.

To visualize physical and functional interactions between the proteins and to determine enriched pathways within the protein set we used the STRING (“Search Tool for Retrieval of Interacting Genes/Proteins”) version 11.0 (34) (https://string-db.org/). The program integrates protein-protein interaction data and functional classification systems of various sources and computes confidence scores for protein interactions. We searched for protein-protein interactions in our dataset using only connections with high confidence using the minimum required interaction score (0.7) and searched within these interactions for clusters of functional interactions. The enriched pathways were found in the Kyoto Encyclopedia of Genes and Genomes (KEGG) pathway database (38), a knowledge base for gene and gene product function systems that connect genomic information and high-level functional information.

### 2.7. Lipid extraction and lipidomics

Semiquantitative lipidomics was carried out to assess the lipid compositions of LDL and Lp(a). To this end, a lipoprotein sample corresponding to 20 µg total protein was spiked with a mixture of internal lipid standards and for lipidomics, the lipids were extracted by a modified Bligh and Dyer extraction method (39). After evaporation of the solvents, the lipids were reconstituted in methanol/chloroform (2:1, v/v) and stored at −20°C.

The lipids were identified and quantified using LC-MS essentially as described previously (40). Chromatographic separation was carried out in gradient mode using a Waters Acquity Ultra Performance LC system equipped with an Acquity BEH C18 1.0 mm × 150 mm (130 Å, 1.7 µM) column. The column temperature was held at 50°C and the flow rate at 0.13 ml/min. Solvent A was acetonitrile/H_2_O (60:40) containing 10 mM ammonium formate and 250 mM ammonium hydroxide; solvent B consisted of isopropanol/acetonitrile (90:10) containing 10 mM ammonium formate and 250 mM ammonium hydroxide. The gradient started from 40% B, changed linearly to 70% B in 10 min, and then linearly to 100% B in 4 min. After 2 min at 100%, B changed to 40% in 3 min, and was retained there for 3 min prior the next injection. The column eluent was directed to the ESI source of a Waters Quattro Premier triple quadrupole mass spectrometer operated in positive ion mode. Phosphatidylcholine (PC) and sphingomyelin (SM) species were detected by MS/MS by scanning for precursors of *m*/*z* 184 and steryl esters (SE) by scanning for precursors of *m*/*z* 369. Since the vast majority of SEs in lipoproteins are esters of cholesterol, we will refer to them as cholesteryl esters (CE) throughout the manuscript. Diacylglycerols (DAG) were detected by selected reaction monitoring (SRM) using transitions relating to a neutral loss of a fatty acyl moiety from the [M + NH_4_]^+^ precursor. Ceramides were detected by SRM using transitions from the [M + H]^+^ precursor to *m*/*z* 264/282. TAGs were detected as [M + NH4]^+^ ions from the full MS scan. The lipids were identified, the ion chromatograms integrated, and the quantities of the individual lipid species calculated using the QuanLynx software (Waters) using curated custom target list.

### 2.8. Fatty acid analysis

For the fatty acid (FA) analysis, lipids were extracted by the Folch method (41). After evaporation of the solvents, the lipids were reconstituted in methanol/chloroform (2:1, v/v) and stored at −20°C.

The FA composition of the particles was analyzed by gas chromatography according to previously published procedures (42). In brief, the extracted lipids were transmethylated by heating with 1% H_2_SO_4_ in methanol under nitrogen atmosphere, and the formed FA methyl esters (including dimethyl acetals DMA, derived from phospholipid alkenyl chains) were extracted with hexane, dried, and concentrated. They were then injected into a GC-2010 Plus gas chromatograph (Shimadzu Scientific Instruments, Kyoto, Japan) with flame-ionization detector for quantification of the FAs. Identification of the FAs was performed using a GCMS-QP2010 Ultra (Shimadzu Scientific Instruments, Kyoto, Japan) with mass selective detector (MSD). Both systems were equipped with Zebron ZB-wax capillary columns (30 m, 0.25 mm ID and film thickness 0.25 µm; Phenomenex, Torrence CA, USA). The FA compositions were expressed as mol% profiles.

### 2.9. Statistical analysis

Normality of the data was analyzed using the Shapiro–Wilk test. Normally distributed data were analyzed by two-tailed unpaired Student's *t*-test for two-group comparisons and one-way ANOVA for comparison of multiple groups with Dunnett's multiple comparisons tests using GraphPad, Prism (version 8.4.3; GraphPad Software, La Jolla, CA). Non-normally distributed data were analyzed by Friedman test or Kruskal-Wallis test with Dunn's *post hoc* test. Correlations were investigated by calculation of the Pearson coefficient and its *p*-value. *p *< 0.05 was considered to be statistically significant.

## 3. Results

### 3.1. Lp(a), and to a lesser extent apo(a), induces transcription of inflammation-associated genes with two clusters around interferon I signaling and IL-10 signaling

**Figure 1:**
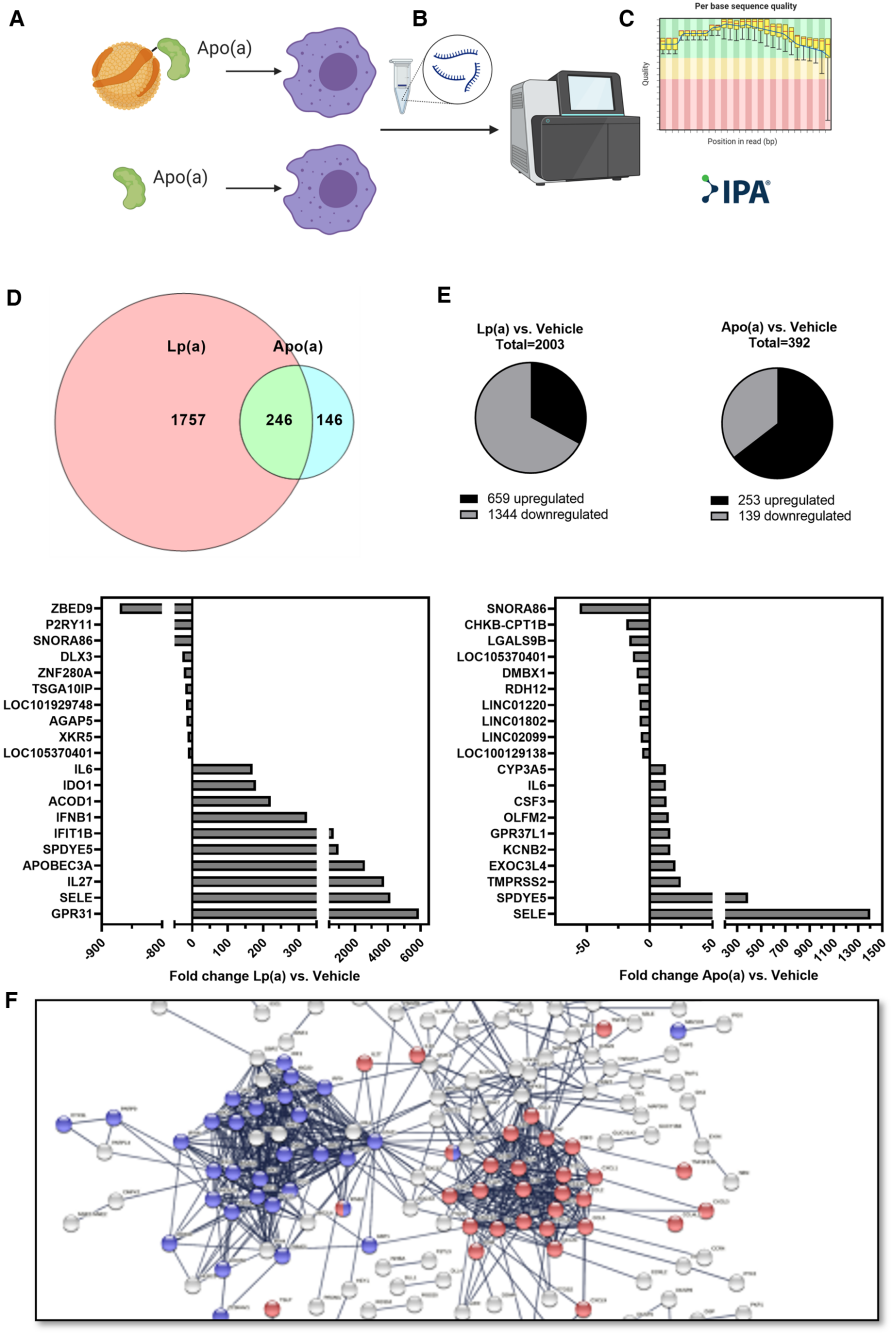
RNA sequencing shows Lp(a) induces inflammatory response in THP-1 macrophages. THP-1 macrophages were starved in serum-free complete medium for 16 h, followed by replacing of the medium with fresh serum-free medium supplemented with equimolar concentrations of Lp(a) and 17 K apo(a) (250 nmol/L, or 191 and 62.5 µg/ml protein, respectively), or no supplement for 6 h. RNA was extracted, and RNA sequencing was performed. The dataset was filtered to contain only genes which have a fold change of at least 2 compared to controls and an Anova score of *p *≤ 0.05. (**A**) The datasets for differentially expressed genes of Lp(a) and apo(a) overlapped by 246 genes. (**B**) For treatment with Lp(a), 2003 genes were expressed differentially, with 659 upregulated and 1,344 downregulated genes. (**C**) Treatment with apo(a) lead to the differential expression of 392 genes of which 253 were upregulated and 193 downregulated. (**D**) The top 10 most downregulated genes for Lp(a) were [protein-encoding genes ZBED9 (Zinc Finger BED-Type Containing 9) and P2RY11 (Purinergic Receptor P2Y11), RNA-encoding SNORA86 (Small Nucleolar RNA, H/ACA Box 86), protein-encoding genes DLX3 (Distal-Less Homeobox 3), ZNF280A (Zinc Finger Protein 280A), and TSGA10IP (Testis Specific 10 Interacting Protein), long non-coding RNA gene LOC101929748 (Uncharacterized LOC101929748), protein-encoding genes AGAP5 (ArfGAP With GTPase Domain, Ankyrin Repeat And PH Domain 5), and XKR5 (XK Related 5), and long non-coding RNA gene LOC105370401 (Uncharacterized LOC105370401). The 10 most upregulated genes are protein-encoding GPR31 (G Protein-Coupled Receptor 31), SELE (Selectin E), IL27 (Interleukin 27), APOBEC3A (Apolipoprotein B MRNA Editing Enzyme Catalytic Subunit 3A), SPDYE5 (Speedy/RINGO Cell Cycle Regulator Family Member E5), IFIT1B (Interferon Induced Protein With Tetratricopeptide Repeats 1B), IFNB1 (Interferon Beta 1), ACOD1 (Aconitate Decarboxylase 1), IDO1 (Indoleamine 2,3-Dioxygenase 1), and IL6 (Interleukin 6). (**E**) For apo(a) the most upregulated genes were protein-encoding SELE (Selectin E), SPDYE5 (Speedy/RINGO Cell Cycle Regulator Family Member E5), TMPRSS2 (Transmembrane Serine Protease 2), EXOC3L4 (Exocyst Complex Component 3 Like 4), KCNB2 (Potassium Voltage-Gated Channel Subfamily B Member 2), GPR37L1 (G Protein-Coupled Receptor 37 Like 1), OLFM2 (Olfactomedin 2), CSF3 (Colony Stimulating Factor 3), IL6 (Interleukin-6), and CYP3A5 (Cytochrome P450 Family 3 Subfamily A Member 5). The 10 most downregulated genes were RNA-encoding genes SNORA86 (Small Nucleolar RNA, H/ACA Box 86), and CHKB-CPT1B [CHKB-CPT1B Readthrough (NMD Candidate)], protein-encoding gene LGALS9B (Galectin 9B), long non-coding RNA gene LOC105370401 (Uncharacterized LOC105370401), protein-encoding genes DMBX1 (Diencephalon/Mesencephalon Homeobox 1), and RDH12 (Retinol Dehydrogenase 12), and long non-coding genes LINC01220 (Long Intergenic Non-Protein Coding RNA 1220), LINC01802 (Long Intergenic Non-Protein Coding RNA 1802), and LINC02099 (Long Intergenic Non-Protein Coding RNA 2099), and pseudogene LOC100129138 (THAP Domain Containing 3 Pseudogene). The up- and downregulation of the genes are expressed as fold change between Lp(a) or apo(a) and control. (**F**) String protein-protein interaction network for the 659 upregulated genes after stimulation with Lp(a) in the RNAseq experiment. The blue cluster comprises proteins belonging to the reactome pathway “Interferon alpha/beta signaling” (R-HSA-909733) and the red clusters belong to “Interleukin-10 signaling” (R-HSA-6783783).

To achieve a global overview of the effect of Lp(a) on inflammatory gene expression in macrophages, we used purified Lp(a) and 17 K recombinant apo(a), and an RNA-seq approach. RNA was harvested and subjected to RNA-seq. The datasets were filtered to contain only genes whose expression, relative to vehicle control, changed at least two-fold. Stimulation with Lp(a) lead to the differential expression of 2,003 genes, of which 659 were upregulated and 1,344 downregulated, and stimulation with apo(a) lead to the differential expression of 392 genes of which 253 were upregulated and 193 downregulated; the data sets overlapped in 246 genes (Figures 1A–C). The 10 most upregulated genes for treatment with Lp(a) were all protein-encoding genes, the top 10 most downregulated genes included protein-encoding genes as well as RNA-encoding and long-non-coding RNA genes (Figure 1D). The top 10 upregulated genes after treatment with recombinant 17 K apo(a) were also all protein-encoding, and the 10 most downregulated genes included RNA-encoding genes, long non-coding RNA genes, as well as pseudogenes (Figure 1E). To visualize physical and functional interactions between the transcripts and to determine enriched pathways within the gene set we used the STRING (“Search Tool for Retrieval of Interacting Genes/Proteins”) version 11.5 and only relationships with the highest confidence score (0.9) were considered, with unconnected nodes removed from the network. STRING integrates protein-protein interaction data and functional classification systems of various sources such as computational prediction, from knowledge transfer between organisms, and from interactions aggregated from other (primary) databases. Based on these data STRING computes confidence scores for protein interactions. Visualization of these interactions for the Lp(a) dataset showed two clusters of upregulated genes (Figure 1F), which were annotated to belong to the reactome (43) pathway “Interferon alpha/beta signaling” (R-HSA-909733) in blue and “Interleukin-10 signaling” (R-HSA-6783783) in red. The whole graphic can be seen in Supplementary Figure SI.

### 3.2. Serum containing Lp(a) induces caspase-1 mediated IL-1 signaling in macrophages in a dose-dependent manner

**Figure 2:**
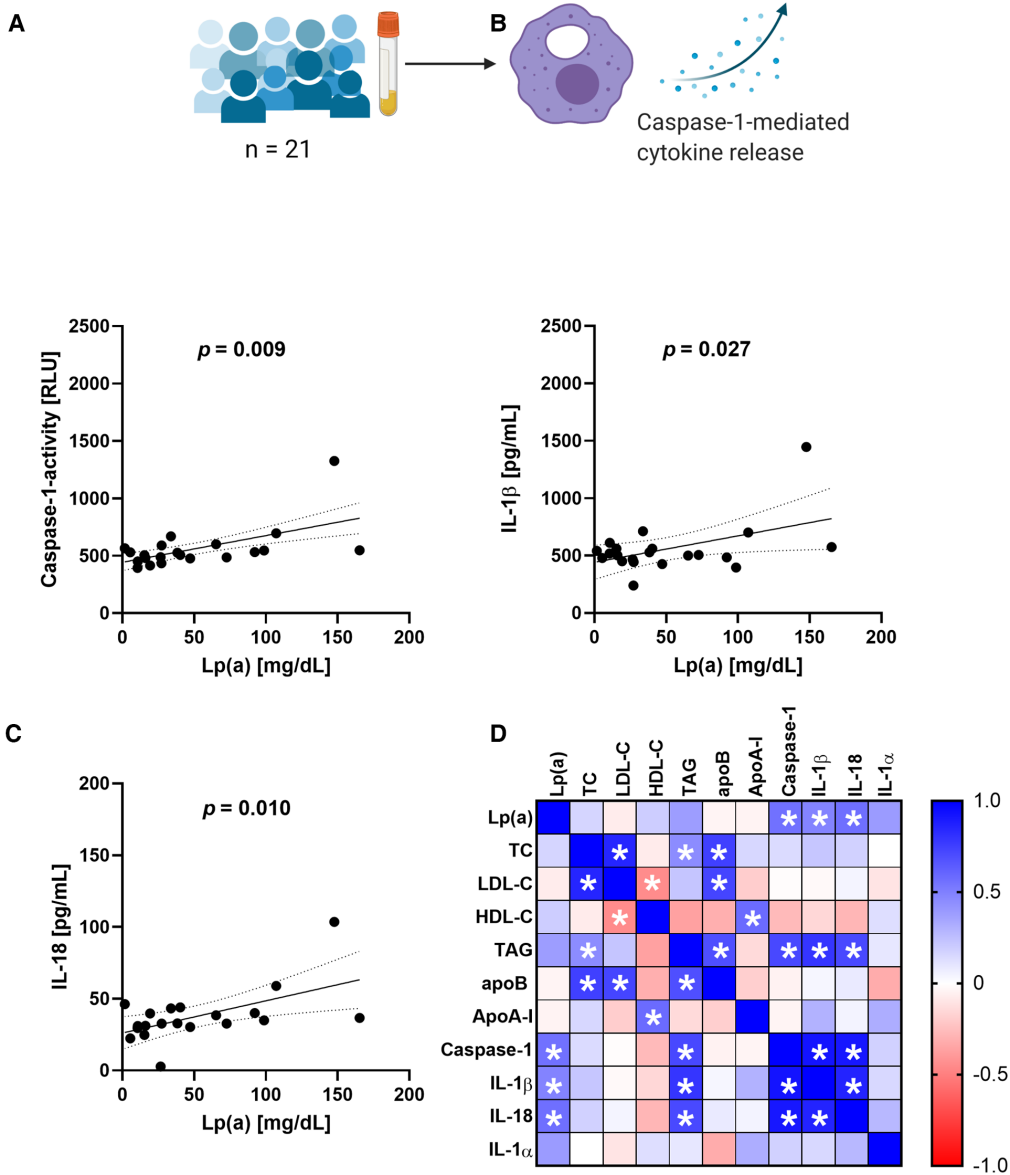
Serum containing Lp(a) induces IL-1 signaling in macrophages. Serum containing various levels of Lp(a) was incubated with THP-1 macrophages for 3 h at 10% v/v, with untreated cells and cells incubated with 4 µM nigericin for 1 h as controls. (**A–C**) The media were collected, and the activity of caspase-1 was determined by GLO-assay, the concentrations of IL-1β and IL-18 were determined by ELISA. These data represent three biological replicates. (**D**) Pearson correlation matrix of the serum values of Lp(a), total cholesterol (TC), LDL-cholesterol (LDL-C), HDL-Cholesterol (HDL-C), triacylglycerols (TAG), ApoB, Apo-AI, as well as the caspase-1 activity and amount of cytokine secretion elicited from the macrophages after incubation with the serum. Significant correlations (*p*-value ≤0.05) are marked with a star, the color gradient visualizes the Pearson correlation coefficients, blue denotes a positive, red a negative correlation.

The RNA sequencing data indicated an involvement of IL-10 signaling and we were curious to see whether this effect can be seen also in THP-1 macrophages treated with serum containing Lp(a). For this purpose, we incubated THP-1-derived macrophages with 10% v/v serum of donors having different levels of circulating Lp(a) (Table 1) as well as the known NLRP3 inflammasome activator nigericin as a positive control. After 3 h of incubation, the conditioned media were collected, and we determined the concentrations of several cytokines belonging to the IL-10 signaling pathway as defined by reactome, namely IL-10, IL-1α, IL-1β, IL-6, IL-8, IL-18, and tumor-necrosis-factor α, (TNF-α). We also measured cytokines related to the IL-10 pathway but not belonging to it such as macrophage migration inhibitory factor (MIF), TNF-related apoptosis-inducing ligand (TRAIL), and IFN-γ, and we determined the activation of caspase-1, which processes IL-1β and IL-18 to their mature secretable forms. We compared these cellular responses with the serum concentrations of Lp(a) as well as other lipid values by Pearson correlation (Figure 2 and Supplementary Figure SII). Lp(a) levels of the sera correlated significantly with caspase-1 activity (*r* = 0.555), as well as the release of IL-1β (*r* = 0.483) and IL-18 (*r* = 0.564) from the macrophages, but not with IL-10, IL-1α, IL-6, IL-8, IL-10, IFN-γ, TNF-α, MIF, or TRAIL (Supplementary Figure II). Serum TAG concentrations correlated with caspase-1 activation and subsequent release of IL-1β, while serum LDL cholesterol (LDL-C) concentrations showed no correlation with any cytokines measured (Figure 2 and Supplementary Figure SII).

**Table 1:** Overview of the clinical parameters of the healthy volunteer donors.

*N*	23
Age, median (25%–75%)	29 (25–43)
Gender, % female	65
BMI, median (25%–75%)	23.8 (21.8–26.2)
TC (mmol/L), mean (SD)	4.1 (0.71)
LDL-C (mmol/L), mean (SD)	2.3 (0.79)
HDL-C (mmol/L), mean (SD)	1.4 (0.44)
TAG (mmol/L), mean (SD)	0.97 (0.64)
Lp(a) (mg/dl), median (25%–75%)	27.3 (16.1–72.6)

TAG, triacylglycerols; TC, total cholesterol; HDL-C, HDL cholesterol; LDL-C; LDL cholesterol.

### 3.3. Lp(a) activates caspase-1 and the subsequent secretion of IL-1 cytokines in macrophages

**Figure 3:**
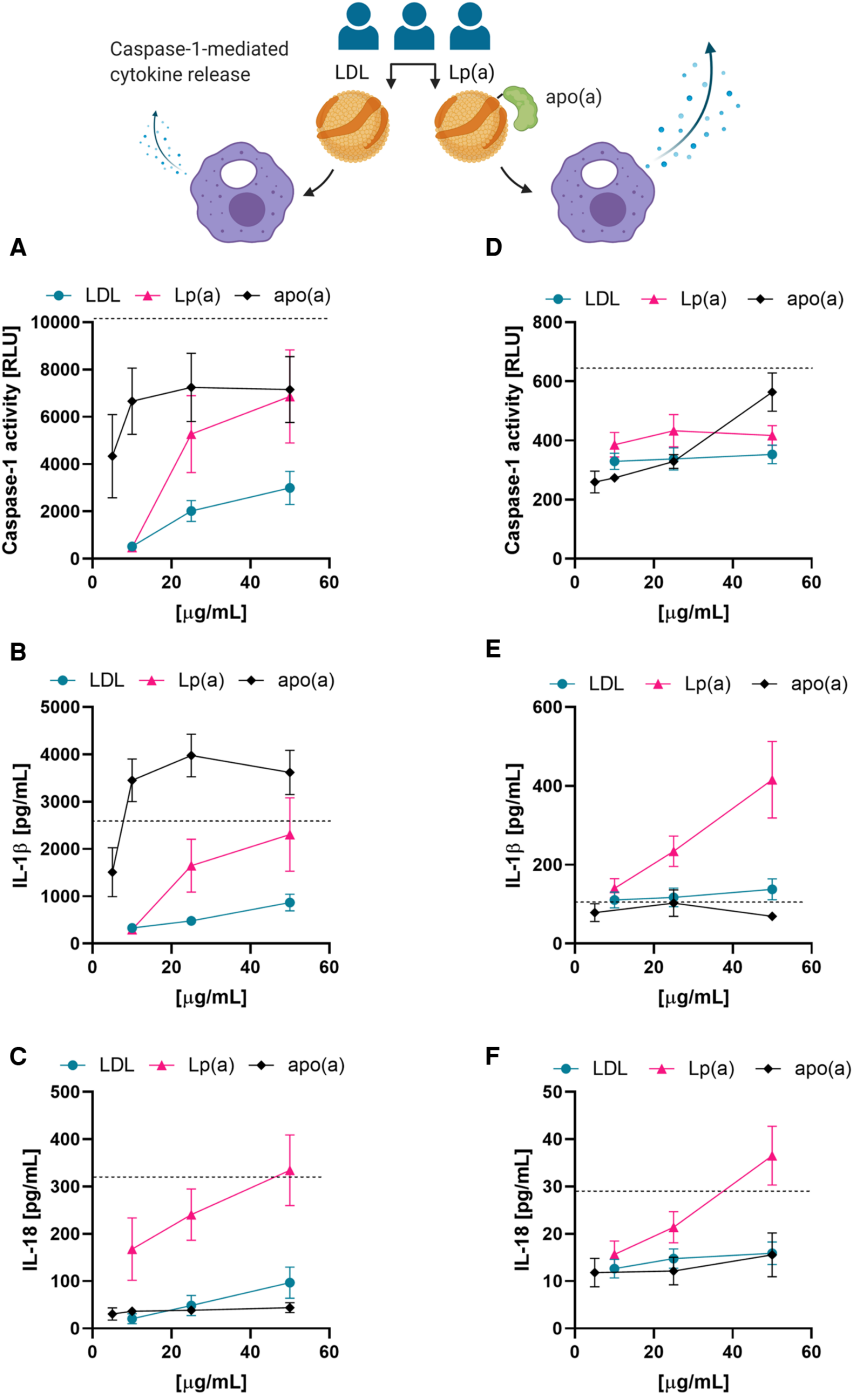
Lp(a) particles and recombinant apo(a) induce caspase-1 activation in macrophages. (**A–C**) THP-1-derived macrophages were incubated in the presence of the indicated concentrations of LDL, Lp(a), or recombinant apo(a) for 3 h. Caspase-1 activity was measured by GLO-assay, IL-18 and IL-1β were measured in the cell culture media by ELISA or Luminex assay. Turquoise is representing the average LDL, pink the average of Lp(a), and black the average of apo(a). The dashed line shows the average of the positive control of the inflammasome activation, 4 µM nigericin for 1 h. The experiment was performed 10 times with 5 paired preparations of the lipoproteins from three different donors and 2 different preparations of apo(a); errors are presented as +/− SEM. (**D–F**) LPS-primed PBMC-derived human macrophages were incubated in the presence of LDL, Lp(a), or apo(a) for 16 h. Caspase-1 activity was measured by GLO-assay, IL-18 and IL-1β were measured from the cell culture media by ELISA or Luminex assay. The dashed line is the average of the inflammasome activation control, 4 µM nigericin for 1 h. The experiments were conducted in cells from 5 individual blood donors and included paired lipoproteins from 3 donors, errors are presented as +/− SEM.

Next, we tested whether the correlations observed between serum Lp(a) concentrations and the upregulation of IL-1 processing could be recapitulated with isolated Lp(a). We thus compared caspase-1 activation in PMA-differentiated THP-1 macrophages treated with LDL or Lp(a) isolated from three different donors as well as recombinant 17 K apo(a) by measuring the activity of caspase-1, as well as the amounts of two of its substrates, IL-1β and IL-18, in the media. As a positive control for NLRP3 inflammasome and thus caspase-1 activation we included 4 µM nigericin for 1 h in all experiments. Both caspase-1 activity and cytokine release responded to a three-hour incubation with LDL, Lp(a), or apo(a) in a dose-dependent manner in THP-1 macrophages, and Lp(a) induced a significantly stronger response than LDL (caspase-1 activity, *p *= 0.0085; IL-1β secretion, *p *= 0.0089; IL-18 secretion *p *= <0.001) (Figures 3A–C). Apo(a) induced an extraordinarily strong caspase-1 activity and secretion of IL-1β even at low concentrations but did not induce secretion of IL-18.

We then examined these trends in primed PBMC-derived macrophages by treating them with LDL, Lp(a), or apo(a) for 16 h with the indicated concentrations (Figures 3D–F). In PBMC-derived macrophages, incubation with the recombinant proteins showed a trend towards induction of caspase-1 activity, but little to no effect on IL-1β or IL-18 secretion. However, Lp(a) showed a slightly stronger induction of caspase-1 activity than LDL, and a very potent induction of IL-1β and IL-18 secretion (Figures 3D–F).

Introduction of caspase-1 inhibitor Z-YVAD-fmk one hour before adding the stimuli resulted in a clear attenuation of caspase-1 activity induction and cytokine release in PBMC-derived macrophages (Supplementary Figure SIII). We also saw an attenuation of the cell response in PBMC-derived macrophages after pre-treatment of the cells with the NLRP3 inflammasome inhibitor MCC950 (Supplementary Figure SIII).

### 3.4. Inflammation-related proteins are enriched in Lp(a) over LDL

**Figure 4:**
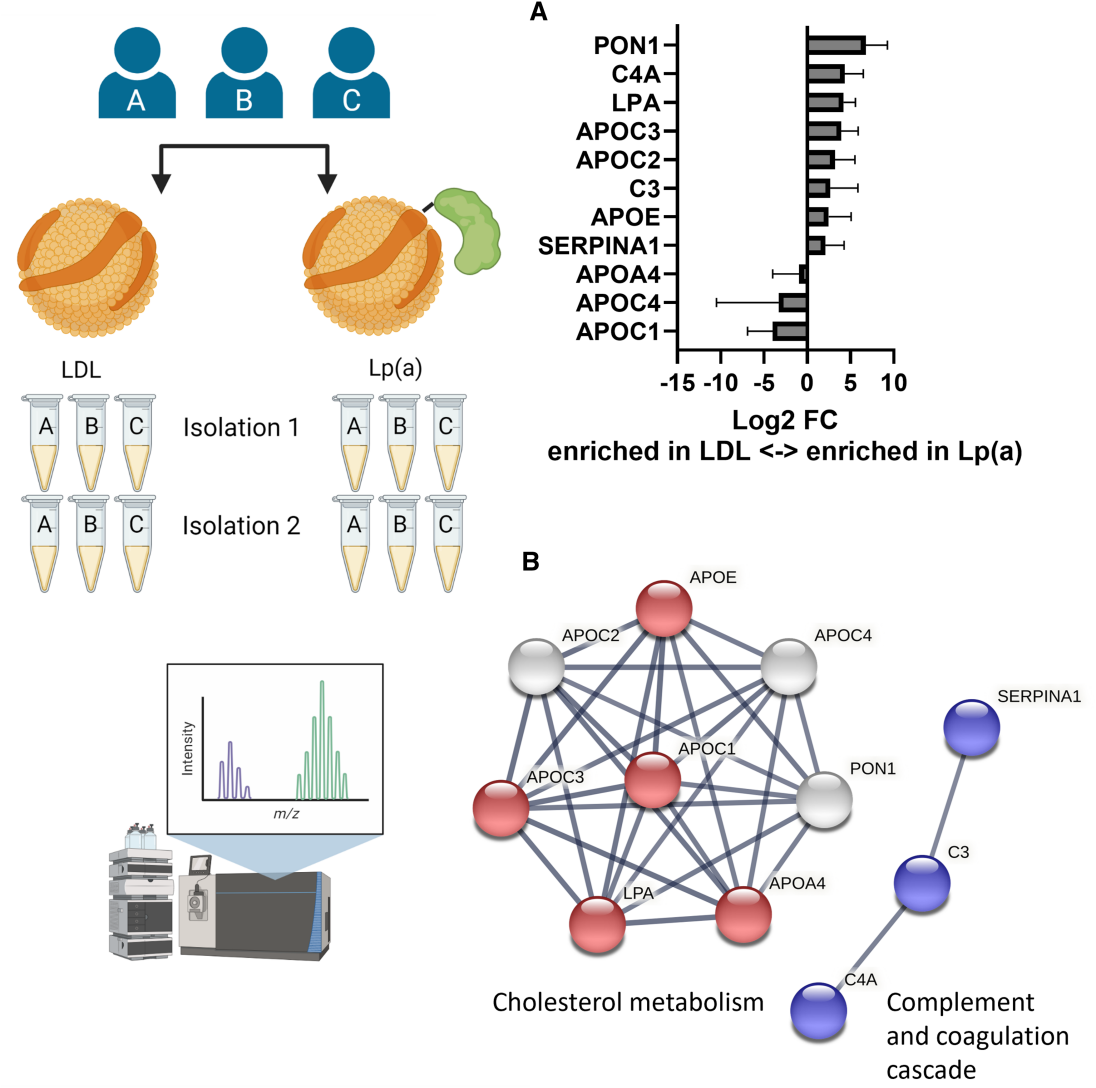
Lp(a) is enriched in proteins associated with cholesterol metabolism and immune response. LDL and Lp(a) were isolated from the same 3 donors at 2 different time points and both sets were analyzed and relatively quantified using LC-MS/MS. After removing known contaminants, proteins identified with a confidence p-value > 0.05, and proteins not identified in all 3 donors, the first isolation yielded 110 proteins and the second 39 proteins. For robustness, only the 11 proteins that were identified in both sets were further analyzed. **(A)** The fold differences were calculated as ratio of the average peak intensities of a protein in Lp(a) over that in LDL from the same donor and are presented as log2 of the fold differences. A log2 value higher than 1 means the protein was at least 2-fold more abundant in Lp(a) than in LDL of the same donor, a value smaller than -1 means the protein was at least 2-fold more abundant in LDL than in Lp(a). **(B)** Visualization of protein-protein interaction clusters of the 11 proteins by String analysis using only connections with high confidence (minimum required interaction score: 0.7). Red nodes belong to the KEGG pathway “Cholesterol metabolism” (hsa04979), blue nodes to the KEGG pathway “ Complement and coagulation cascades” (hsa04610). [ApoA4 (APOA4), apoC1 (APOC1), apoC2 (APOC2), apoC3 (APOC3), apoC4 (APOC4), apoE (APOE), complement C3 (C3), complement C4-A (C4A), apo(a) (LPA), serum paraoxonase/arylesterase 1 (PON1), alpha-1-antitrypsin (SERPINA1)].

To elucidate whether Lp(a) contains, in addition to apo(a), other proteins that have the potential to induce inflammasome activation in macrophages, LDL and Lp(a) were isolated from the same three donors on two separate occasions, and both sets of preparations were analyzed and relatively quantified using LC-MS/MS (Figure 4A). After removing known contaminants commonly found in proteomics samples such as keratins, proteins identified with a confidence *p*-value >0.05, and proteins not identified in all three donors, the first set yielded 110 and the second 39 proteins (Supplementary Table SI). These were normalized setting apoB to be 1, as both LDL and Lp(a) contain exactly one copy (Supplementary Table SI). For robustness, only the 11 proteins that were identified in both sets were further analyzed. Of these, apoA4 (log2FC = −0.91), apoC1 (log2FC = −4.00), and apoC4 (log2FC = −3.30) were enriched in LDL, and apoC2 (log2FC = 3.21), apoC3 (log2FC = 3.92), apoE (log2FC = 2.49), complement C3 (C3, log2FC = 2.67), complement C4A (C4A, log2FC = 4.33), apo(a) (LPA, log2FC = 4.2), serum paraoxonase/arylesterase 1 (PON1, log2FC = 6.76), and alpha-1-antitrypsin (SERPINA1, log2FC = 2.1) were enriched in Lp(a) (Figure 4B). String analysis showed two clusters in this dataset, belonging to the KEGG (38) pathway “Cholesterol metabolism” (ApoA4, apoC1, apoC3, apoE, apo(a)), and “Complement and coagulation cascades” (C3, C4A, SERPINA1) (Figure 4C). Proteins belonging to the complement and coagulation cascades are highly enriched in Lp(a).

### 3.5. Lp(a) is relatively deficient in polyunsaturated fatty acids (PUFAs)

**Figure 5:**
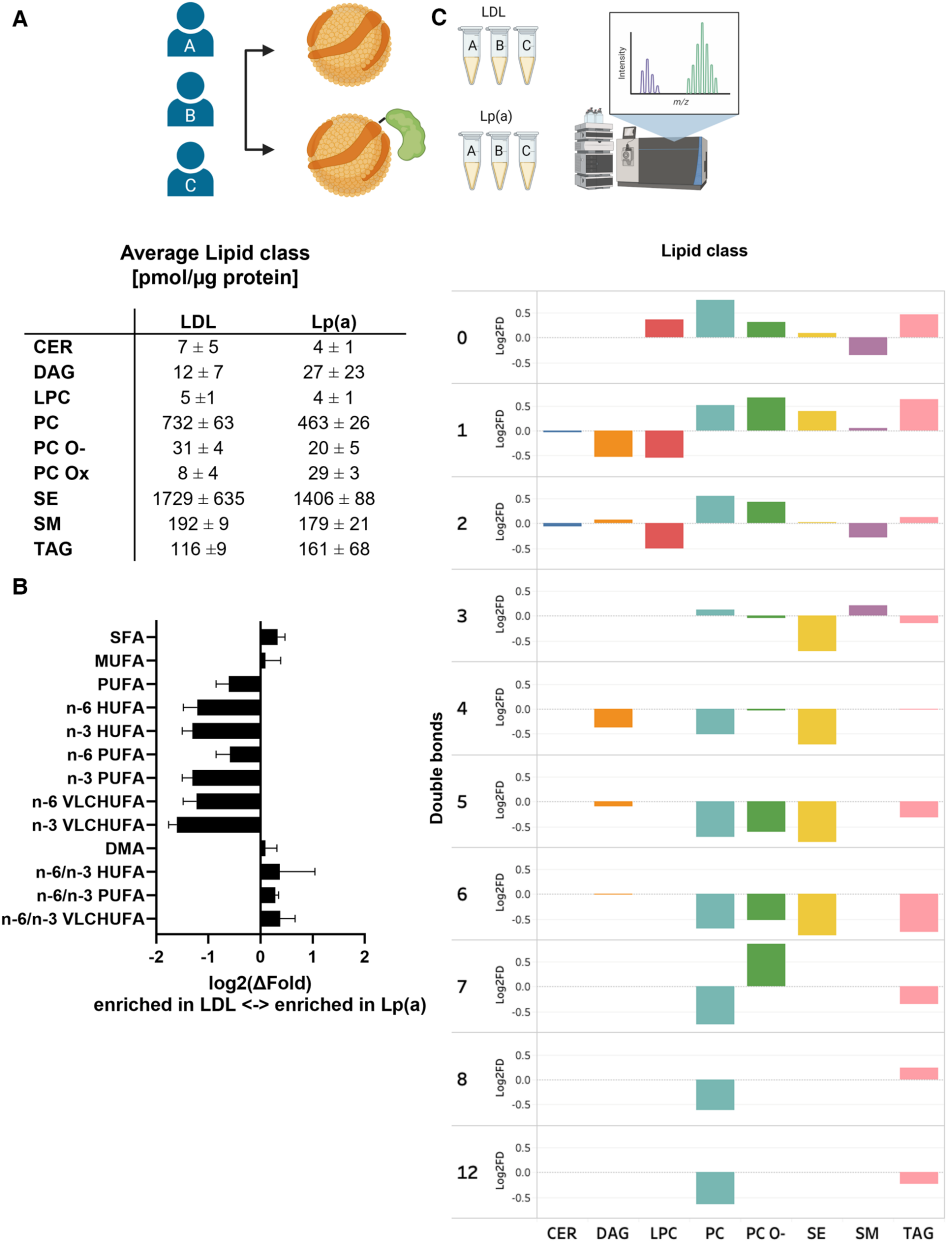
When compared to LDL, Lp(a) is depleted of PUFAs which also have higher n-6/n-3 ratio. LDL and Lp(a) were isolated from the same 3 donors and analyzed using LC-MS. (**A**) Global lipid class profiles (values are pmol/µg protein). The classes are ceramides (CER), diacylglycerols (DAG), lysophosphatidylcholines (LPC), phosphatidylcholines (PC), oxidized phosphatidylcholines (PC-Ox), ether-linked phosphatidylcholines (PC-O), steryl esters (mainly cholesteryl esters, CE), sphingomyelins (SM), and triacylglycerols (TAG). (**B**) Differences between Lp(a) and LDL as the average log2 of the fold difference for structural categories of the fatty acid and alkenyl chains in the total lipids. The categories are saturated (SFA), monounsaturated (MUFA), polyunsaturated (PUFA), very long chain highly unsaturated (VLCHUFA, ≥20 carbons and ≥4 double bonds) and alkenyl chains (DMA, detected as dimethyl acetal derivatives). The PUFAs and VLCHUFAs were divided into the n-6 and n-3 structural families based on the double bond positions in the chain, and additionally, the n-6/n-3 ratios were calculated. (**C**) A comparison of relative lipid abundances (mol% per lipid class) in Lp(a) and LDL particles from each individual donor was carried out. The average log 2 of the fold difference is plotted for each lipid class against the number of double bonds in the lipid acyl chains. A negative value signifies an enrichment in LDL, and a positive value an enrichment in Lp(a).

We next analyzed and compared the lipid class and species compositions of Lp(a) and LDL of the three donors using LC-MS. The proportions of different lipid classes of Lp(a) and LDL were similar, with CEs, PCs, SMs, and TAGs dominating the lipid profile (Figure 5A and Supplementary Table SII). Nevertheless, the PC content of Lp(a) particles was lower than in LDL. The contents of SM in Lp(a) and LDL particles did not differ from each other. As proinflammatory OxPLs have been suggested to be major contributors to the atherogenicity of Lp(a) (44, 45), we were interested to compare those between Lp(a) and LDL samples. We detected multiple low-abundance signals that, based on their *m*/*z*-value, retention time and fragmentation properties, could correlate to oxidized PCs. Such species were increased 3-fold in Lp(a) vs. LDL (Figure 5A).

When looking at the individual lipid species comprising the different lipid classes, remarkable and consistent differences between the two particle types emerged. Out of 184 measured lipid species, 18 were significantly different between the particles. Most of these species were polyunsaturated PC molecules, which were depleted in Lp(a). Furthermore, a pairwise comparison of LDL and Lp(a) particles from the same donors revealed a systematic underrepresentation of lipid species with 3–12 double bonds in Lp(a) particles indicating that Lp(a) is generally poor in PUFA, when compared to LDL (Figure 5C and Supplementary Table III).

To further characterize the observed differences, we analyzed the total FA profiles of Lp(a) and LDL using gas chromatography and then assessed the n-6/n-3 ratios. Clearly, while Lp(a) was relatively depleted in PUFAs, especially the n-3 PUFAs, it had a much higher n-6/n-3 PUFA ratio than LDL (Figure 5B). We repeated the analyses with another set of isolated lipoproteins from all three donors and the trend of relative PUFA depletion of Lp(a) remained robust (data not shown).

## 4. Discussion

In this study we investigated the effects of Lp(a) and apo(a) on macrophage gene expression using RNA sequencing and contrasted the potential of isolated Lp(a) vs. LDL in inducing inflammation in macrophages. We also characterized by proteomic and lipidomic analysis the molecular profile of Lp(a) and LDL particles isolated from the same three donors with elevated Lp(a) levels. These unprecedented studies provide novel insights into the molecular basis of Lp(a) pathogenicity while underscoring the unique metabolic origins and characteristics of Lp(a).

In the first step we performed RNA sequencing on THP-1 derived macrophages stimulated with Lp(a) and recombinant apo(a) to gain an overview of the induced processes. Both Lp(a) and recombinant apo(a) induced the upregulation of genes belonging to the reactome IL-10 signaling pathway. We analyzed the secretion of a set of members of this pathway together with related cytokines from macrophages treated with serum from donors with varying levels of plasma Lp(a). All the study participants were relatively young and healthy and not under any medication that would influence inflammation, such as statins, showing that Lp(a) is pro-inflammatory already in young healthy individuals without confounding effects of existing ASCVD.

To our surprise it was not IL-10, but IL-1β, IL-18, and their processing enzyme caspase-1 that all showed a significant correlation with the Lp(a) level in the serum (Figure 2 and Supplementary Figure SII). The fact that the THP-1 macrophages are to some degree primed after PMA differentiation and have induced expression of genes involved in IL-1 family signaling including IL-1β might have masked the true induction of these genes by Lp(a) and apo(a) in the RNA sequencing experiment (46); however, expression of IL-1β was markedly upregulated (13.4-fold upregulated after stimulation with Lp(a) and 5.7-fold upregulated after stimulation with recombinant apo(a)), but components of the NLRP3 inflammasome and IL18 were not induced as expected in primed macrophages.

Serum TAG concentrations correlated with caspase-1 activation and subsequent release of IL-1β which has been shown before (47), and as a novel finding they also significantly correlated with release of IL-18. The release of IL-8 was less pronounced as expected based on previous literature (21, 48), however the RNA-seq data showed that CXCL8 mRNA was increased ∼18-fold by Lp(a) and ∼5-fold by 17 K apo(a). In the stimulation with plasmas containing Lp(a) we observed a slight trend towards increased expression of IL-8 but note the variability in the response to different plasmas, that may have obscured an increase. The plasmas may also have contained factors that impact the ability of Lp(a) to regulate expression of CXCL8.

Lp(a) has been shown to induce IL-8 (21), IL-6 and TNF (44), but it has not been shown to directly induce caspase-1 mediated IL-1 family signaling *in vitro*. We next incubated THP-1 macrophages with isolated Lp(a) particles in amounts up to 50 µg/ml [5 mg/dl; well under the pathophysiological levels of 30–50 mg/dl (49)], which induced caspase-1 activation in a dose-dependent manner that was significantly higher than for LDL isolated from the same donors at the same time (Figures 3A–C). This clarifies that Lp(a), and not other serum components from patients with Lp(a) hyperlipidemia, is responsible for inducing caspase-1-mediated inflammasome activation in macrophages. Lp(a) also induced a similar, albeit lower, response in primary macrophages, in line with a lower response to the positive control (Figures 3D–F). We confirmed in primary macrophages the caspase-1 activation using both the caspase-1 inhibitor Z-YVAD-fmk as well as upstream the NLRP3 inflammasome inhibitor MCC950 (Supplementary Figure SIII). Also, recombinant 17 K apo(a) activated caspase-1 and IL-1β secretion even at low concentrations in THP-1 macrophages, but not in PBMC-derived macrophages, and it did not induce secretion of IL-18 (Figure 3). There are distinct regulatory mechanisms controlling proinflammatory cytokines IL-18 and IL-1β [Reviewed in (50)]; IL-18 is additionally activated via interferon signaling, which is abundant in atherosclerosis (51–53) and is prominent as a cluster in the RNA sequencing data (Figure 1F). The difference in the magnitude of the inflammatory potential of Lp(a) in THP-1 and PBMC-derived macrophages is most likely due to THP-1 macrophages being much more M1-like, while PBMC-derived macrophages represent a more varied pool of macrophage polarizations (54, 55).

The above-discussed results indicate strong pro-inflammatory properties of Lp(a) in terms of caspase-1-activation and IL-1β and IL-18 secretion. This pathway involves the activation and assembly of the NLRP3 inflammasome and subsequent activation of caspaes-1. Activated caspase-1 facilitates the cleavage and secretion of pro-inflammatory IL-1 family cytokines such as IL-1β and IL-18 (50). Our results are in line with the plethora of data implicating IL-1 signaling in atherogenesis [reviewed in (50)]. For one, CANTOS (Canakinumab Anti-Inflammatory Thrombosis Outcomes Study) demonstrated the importance of this pathway and the therapeutic potential of addressing IL-1β secretion (56). The NLRP3 inflammasome is strongly expressed in atherosclerotic lesions (57), and is activated by cholesterol crystals (32, 58) as well as various forms of modified LDL, such as oxidized LDL (59), oxidized LDL-immune complexes (60), or electronegative LDL (61). Importantly, apoB-containing lipoproteins isolated from human carotid atherosclerotic plaques induce inflammasome and caspase-1 activation (62). The exact molecular triggers for inflammasome and subsequent caspase-1 activation by different modified LDL species are not yet fully understood, but it is thought that unesterified cholesterol derived from phagocytosed LDL particles form cholesterol crystals, which in turn activate the NLRP3 inflammasome (63). Inflammasome activation subsequently contributes to foam cell formation, a hallmark of atherosclerosis (2, 64, 65). Our data raise the possibility that Lp(a) in its native circulating form that harbors oxPL also serves to activate the NLRP3 inflammasome.

To elucidate the cause of the pro-inflammatory effects of Lp(a) we performed structural analyses of the particles compared with LDL isolated from the same donors at the same time. Proteomics analysis of LDL and Lp(a) derived simultaneously from the same three donors at two individual time points identified 11 proteins that are robustly found in lipoprotein isolations of the three donors. Of these, apoC2, apoC3, apoE, complement C3, complement C4A, apo(a), serum paraoxonase/arylesterase 1, and alpha-1-antitrypsin were more abundant in Lp(a) than in LDL (Figure 4B). The proteins belonged to two clusters; proteins associated with inflammation were α-1 antitrypsin and complement components C3 and C4A. Of these, α-1 antitrypsin has been implicated in atherosclerosis, but in a protective function (66), and complement C3 products and their cell receptors have been detected by immunohistochemistry in areas with atherosclerotic lesions of different severity in human arteries (67–69). The second cluster with proteins belonging to cholesterol metabolism includes apolipoproteins as well as serum paraoxonase/arylesterase 1. ApoA4, apoC1, and apoC4 were the only proteins more abundant in LDL particles which was surprising as all three have been considered to be enriched in Lp(a) in studies by another group, albeit not all within the same study (70, 71). ApoA4 is considered to be anti-atherogenic; it is required for efficient activation of lipoprotein lipase by apoC2 (72) and is a potent activator of the lecithin:cholesterol acyltransferase (73, 74). ApoC1 is an inhibitor of lipoprotein binding to their respective receptors, and it downregulates lipoprotein lipase, hepatic lipase, phospholipase A2, cholesteryl ester transfer protein, and activates lecithin:cholesterol acyltransferase (74, 75). It further binds free FAs and reduces their intracellular esterification. Not much is known about apoC4, but human apoC4 transgenic mice were shown to be hypertriglyceridemic compared to non-transgenic controls, however, the plasma triglycerides were present mainly in VLDL, not in LDL (76).

PUFAs are precursors to various lipid mediators: n-3 PUFAs, including eicosapentaenoic acid and docosahexaenoic acid, serve as precursors to a range of metabolites which are considered anti-inflammatory or pro-resolving, while n-6 PUFAs, including arachidonic acid, yield pro-inflammatory mediators (77). Lipidomic comparison of Lp(a) and LDL revealed that PUFA content was clearly lower in all lipid classes of Lp(a) (Figure 5B), and due to their low total n-3 PUFA content, the n-6/n-3 PUFA-ratio was markedly higher than that of LDL (Figure 5C), suggesting that lipids in these particles are derived from different lipid pools. This in turn suggests that Lp(a) particle biogenesis is distinct from that of LDL. This is supported by recent studies indicating different mechanisms for the formation of Lp(a)-apoB and LDL-apoB (78–80), as well as an intracellular interaction between apo(a) and apoB that controls the rate of Lp(a) biogenesis (81). On the other hand, it is possible that the apo(a) modulates interactions with lipoprotein-modifying proteins such as lipoprotein lipase, phospholipid transfer protein or cholesteryl ester transfer protein, resulting in the observed differences. Indeed, a substantial portion of the CEs in LDL arise from reverse cholesterol transport, further underscoring the distinct metabolism of Lp(a). Nevertheless, the implications of Lp(a) lipid composition on its inflammatory potential remain obscure at present. However, the fact that Lp(a) had relatively higher content of pro-inflammatory n-6 PUFA with a reduction of anti-inflammatory or pro-resolving n-3 PUFAs could contribute to the pro-inflammatory nature of Lp(a) (82–84).

When comparing the overall lipid levels of Lp(a) and LDL, the ratio of surface lipids (phospholipids and SM) to core lipids (cholesteryl esters and TAGs) is lower in Lp(a) than in LDL. This suggests that Lp(a) particles are slightly larger than LDL particles. In addition to the potential difference in particle size, the proportion of SM in Lp(a) was much higher than in LDL. These differences could play a role in the different effect of Lp(a) and LDL on macrophages.

We also detected MS-signals potentially attributable to various oxPLs, including POVPC, in Lp(a) samples, but not in LDL from the same donors. These signals were present in low abundance, and likely correspond to oxPL non-covalently associated with apo(a), apoB, or the lipid moiety of the particle. Notably, these signals would not correspond to the oxPL which has been found to be covalently associated with KIV type 10 in apo(a) (21, 85), as the latter species would not be extracted from the particle under these conditions. POVPC, when added to macrophages to yield at a final concentration of 10 µM, has been shown to upregulate cytokine genes (26). When administered to cultured mouse macrophages to reach a final concentration of >5 mg/dl (>8.4 mM) levels, POVPC has been shown to directly induce NLRP3 inflammasome activation (86). POVPC and other oxPLs likely contribute to the inflammatory nature of Lp(a), an idea underscored by the markedly enhanced pro-inflammatory potency of Lp(a) compared to apo(a) alone revealed by our RNA-seq data. It is therefore imperative to further characterize these lipid mediators.

The main question remaining is the exact mechanism in which Lp(a) induces pro-inflammatory responses from macrophages and other cells. Previous we found that Lp(a) and apo(a) stimulated β-catenin nuclear translocation in endothelial cells through a pathway involving Akt as well as Src and Rho/Rho kinase (13). In that study, the apo(a) variant lacking the KIV10 strong lysine binding site—and thus bound oxPL—had no effect on this pathway, implicating either the oxPL or the strong LBS in activating this pathway. In another study, we found that stimulation of *CXCL8* (IL-8) gene expression by Lp(a) was associated with NFκB activation through CD36 and TLR2 receptors acting through MAP kinases, Jun N-terminal kinase and ERK1/2 (21). These receptors are known targets of oxPL, again implicating the oxPLs as a major component in Lp(a) pathogenicity. Potentially, the NFκB activation coincidentally leads to activation of IL-1 signaling as this is the result of the priming signal in the IL-1 pathway, leading to the expression of NLRP3 inflammasome components as well as pro-IL-1β (50).

In conclusion, we have documented that, compared to LDL, Lp(a) more strongly induces inflammatory responses in macrophages, both as the isolated particle and within human serum. These observations likely arise from the presence of proteins with inherent pro-inflammatory properties, as well as a distinct lipid composition that features a lower abundance of PUFAs yet a higher ratio of pro-inflammatory n-6 PUFAs to n-3 PUFAs. Our findings warrant extending our analysis to include a larger pool of donors to further evaluate all these differences. Such analyses may identify Lp(a)-associated biomarkers that amplify the risk associated with Lp(a).

### 4.1. Limitations of the study

The main limitation of the study is the sample size, we would have preferred a larger cohort of healthy individuals with varying Lp(a) plasma levels, and ideally would have had the means to isolate LDL and Lp(a) at the same time from a large number of individuals with elevated Lp(a).

The isolated particles were not tested for LPS as it has never been found, however, such a contamination would be found in both LDL and Lp(a) fractions.

### 4.2. Translational perspective of findings

Our findings implicate a role of IL-1 signaling in Lp(a)-driven vascular inflammation. However, this additional novel information is not sufficient alone to support antiatherogenic interventions via IL-1β inhibition, as the CANTOS trial indicated that a general reduction of IL-1β also carries the risk of potentially fatal infections (87). Yet, it is important to know which inflammatory pathways are involved in the Lp(a)-driven disease pathology. Targeting residual inflammatory risks in atherosclerotic cardiovascular diseases is currently being investigated in several trials (88, 89). Another aspect, and maybe a safer approach, is to strengthen the resolution of inflammation in cardiovascular diseases (2). Together with the general lowering of the inflammation-inducing lipoproteins, among them the Lp(a) (90), an increased resolution of IL-1β-mediated vascular inflammation would allow an additional tool when attempting to reduce the risk of cardiovascular disease in people with an elevated level of Lp(a).

## Data Availability

The proteomics and lipidomics data can be found as tables in the supplemental materials. The RNAseq data discussed in this publication have been deposited in NCBI's Gene Expression Omnibus (91) and are accessible through GEO Series accession number GSE226759 (https://www.ncbi.nlm.nih.gov/geo/query/acc.cgi?acc=GSE226759).
