# Supplementary material for: Lipoprotein(a) induces caspase-1 activation and IL-1 signaling in human macrophages
Source: Front Cardiovasc Med. 2023 May 24;10:1130162. doi: 10.3389/fcvm.2023.1130162 (PMC10244518; doi:10.3389/fcvm.2023.1130162)
Supplement: Supplementary file 1 [file Datasheet1.pdf]

Lipoprotein(a) induces caspase-1 activation and IL-1 signaling in human macrophages

Martina B. Lorey [1,4], Amer Youssef [2], Lauri Äikäs [1], Matthew Borrelli [3], Martin Hermansson [1], Julia M. Assini [2,7], Aapeli Kemppainen [1], Hanna Ruhanen [4,5], Maija Ruuth [1,4], Sampsa Matikainen [6], Petri T. Kovanen [1], Reijo Käkelä [4,5], Michael B. Boffa [2,7], Marlys L. Koschinsky [2,3], and Katariina Öörni [1,4].

1. Atherosclerosis Research Laboratory, Wihuri Research Institute, Helsinki, Finland.
2. Robarts Research Institute, Schulich School of Medicine & Dentistry, The University of Western Ontario, London, Ontario, Canada
3. Department of Physiology & Pharmacology, Schulich School of Medicine & Dentistry, The University of Western Ontario, London, Ontario, Canada
4. Molecular and Integrative Biosciences, Faculty of Biological and Environmental Sciences, University of Helsinki, Helsinki, Finland
5. Helsinki University Lipidomics Unit (HiLIPID), Helsinki Institute of Life Science (HiLIFE) and Biocenter Finland, Helsinki, Finland
6. Helsinki Rheumatic Disease and Inflammation Research Group, University of Helsinki and Helsinki University Hospital, Helsinki, Finland.
7. Department of Biochemistry, Schulich School of Medicine & Dentistry, The University of Western Ontario, London, Ontario, Canada

Corresponding author: Katariina Öörni, Tel: +358 96814133, Fax: +358 9637476, Email: kati.oorni@wri.fi

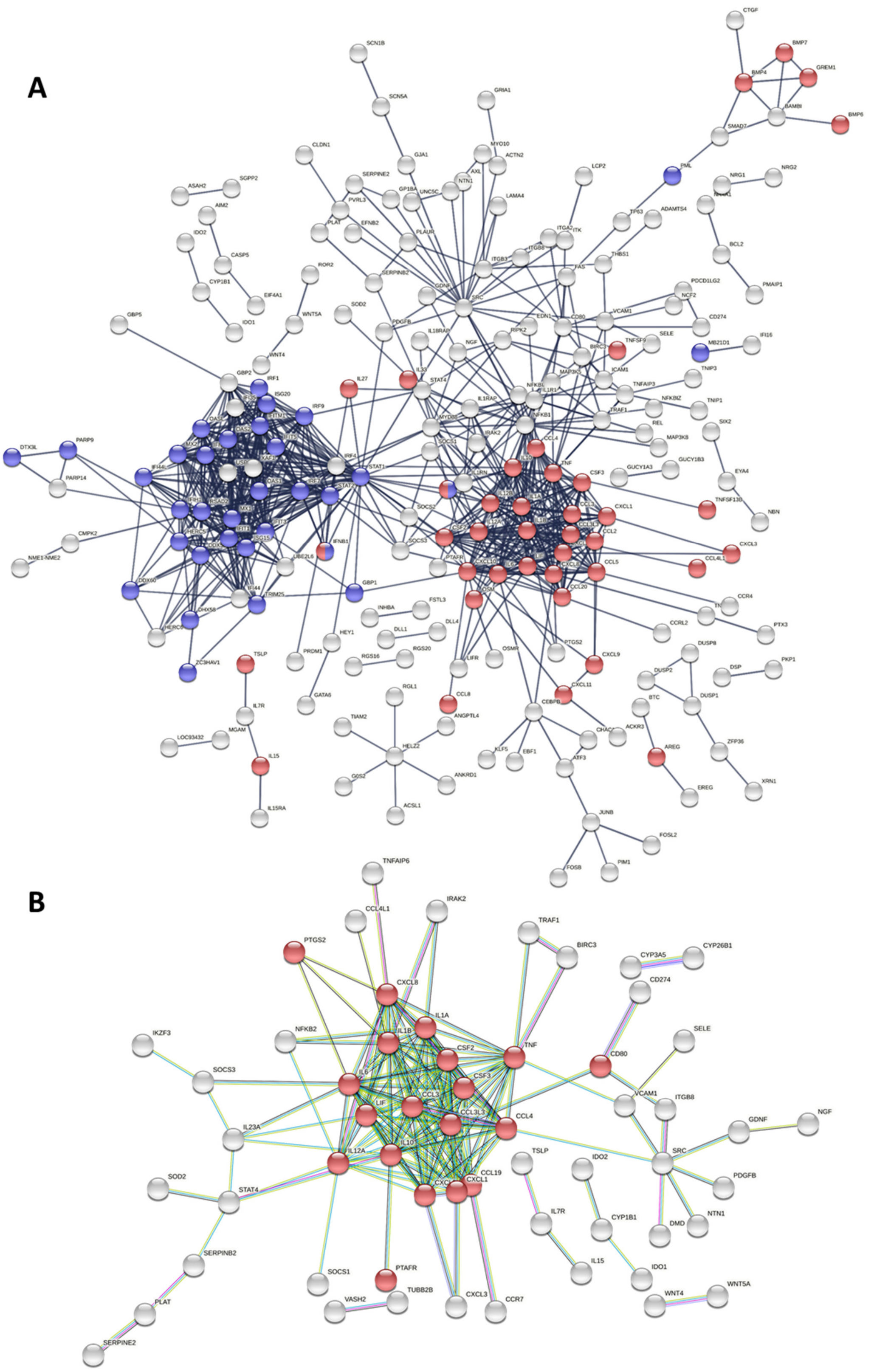

Supplemental figure I. String protein-protein interaction networks. A) Protein-protein interaction network for the 659 upregulated genes after stimulation with Lp(a) in the RNAseq experiment, B) protein-protein interaction network for the 253 upregulated genes after stimulation with recombinant apo(a) in the RNAseq experiment. The blue cluster are proteins belonging to the reactome pathway "Interferon alpha/beta signaling" (HSA-909733) and the red clusters belong to "Interleukin-10 signaling" (HSA-6783783).

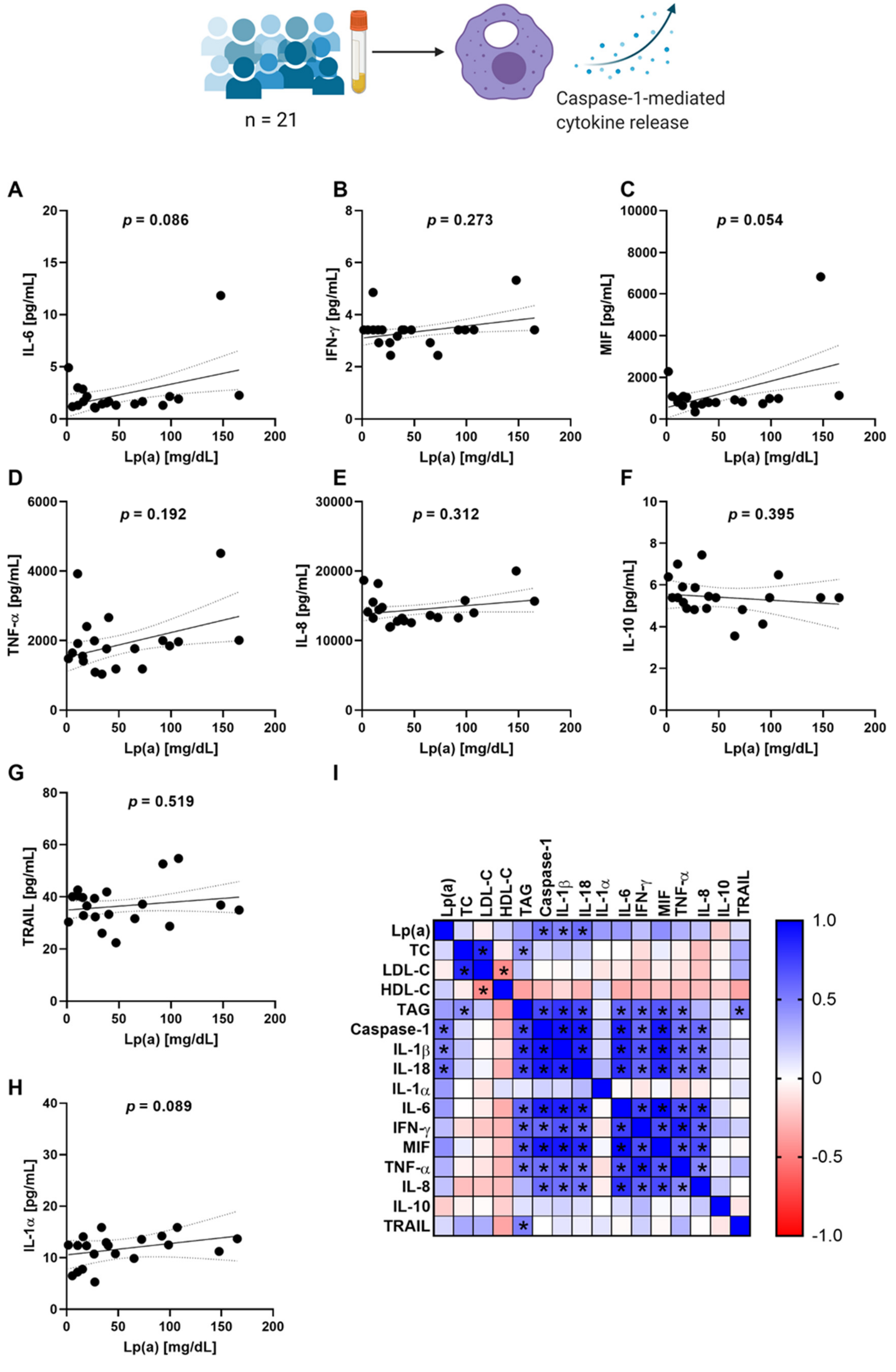

Supplemental figure II. Plasma containing Lp(a) induces cytokine release.
Plasma containing various levels of Lp(a) was incubated with macrophages for 3 hours at 10 % v/v, with untreated cells and cells incubated with 4 µM nigericin for 1h as controls. A-H) The media were collected, and the concentrations of IL-6, IL-8, IL-10, IFN-γ, TNF-α, MIF, TRAIL, and IL-1α were determined by LUMINEX assay. The trendline is given with the 95% CI. I) The Spearman correlation matrix for all blood lipid and cytokine release measurements, significant correlations with a p-value of correlation ≤ 0.05 are marked with a star, the color code represents the degree of correlation between the data sets. These data are based on 3 independent experiments.

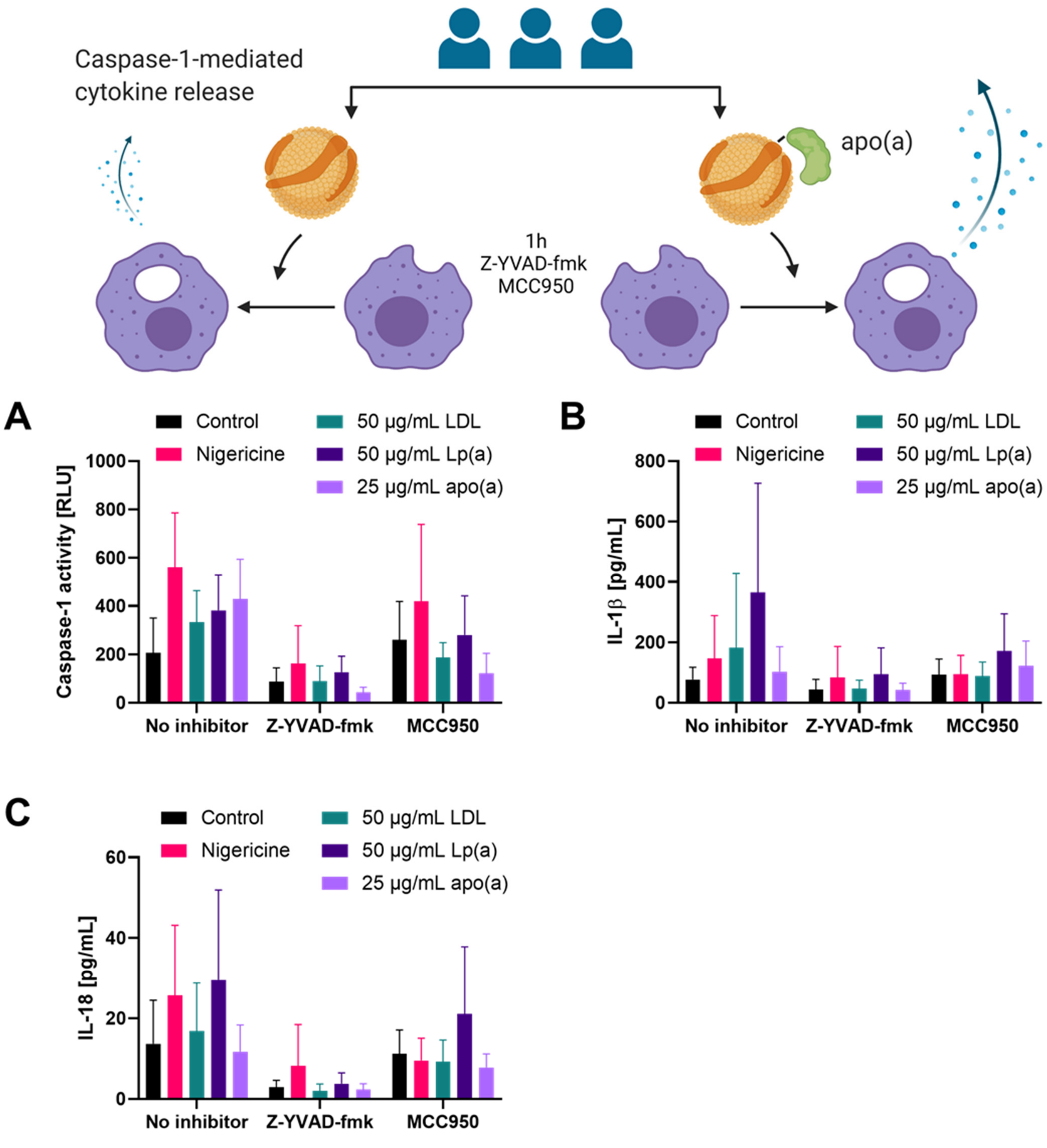

Supplemental figure III. Inhibition of caspase-1 and NLRP3 attenuates cellular response to apo(a) in human macrophages.

(A-C) PBMC-derived macrophages were left untreated or treated for 1h with either 25 µM of the caspase-1 inhibitor Z-YVAD-fmk or 1µM of the NLRP3 inflammasome inhibitor MCC950. After that the cells were incubated with 50 µg/mL LDL or Lp(a), or 25 µg/mL recombinant apo(a) for 3h or 4 µM nigericin for 1h; the experiment was performed 3 times with LDL and Lp(a) isolated from 3 donors. Caspase-activity was measured by GLO-assay, IL-18 and IL-1β were measured from the cell culture media by ELISA assay. Error bars are presented as +/- SD.

Accession	To	Description	Log2FC DA_1	Log2FC DB_1	Log2FC DC_1	Log2FC DA_2	Log2FC DB_2	Log2FC DC_2
P01023	A2M	Alpha-2-macroglobulin	2.67	3.58	-0.73	-	-	-
Q9H9F9	ACTR5	Actin-related protein 5	-6.18	-0.35	-7.37	-	-	-
Q9Y5C1	ANGPTL3	Angiopoietin-related protein 3	-3.37	-3.08	-4.76	-	-	-
P02743	APCS	Serum amyloid P-component	2.71	3.78	5.73	-	-	-
Q9HDC9	APMAP	Adipocyte plasma membrane-associated protein	3.57	3.70	7.57	-	-	-
P02652	APOA2	Apolipoprotein A-II	2.47	7.09	-2.31	-8.59	-4.68	-1.15
P06727	APOA4	Apolipoprotein A-IV	0.14	4.74	-1.83	-4.06	-1.85	-2.62
Q6Q788	APOA5	Apolipoprotein A-V	2.82	4.89	2.10	-	-	-
P04114	APOB	Apolipoprotein B-100	-	-	-	0.00	0.00	0.00
P02654	APOC1	Apolipoprotein C-I	-4.28	1.14	-4.13	-7.15	-6.16	-3.41
P02655	APOC2	Apolipoprotein C-II	4.06	6.71	0.90	0.38	3.94	3.29
P02656	APOC3	Apolipoprotein C-III	4.84	5.43	0.68	2.28	5.21	5.08
P55056	APOC4	Apolipoprotein C-IV	0.06	4.51	0.58	-15.56	-7.62	-1.79
P05090	APOD	Apolipoprotein D	2.02	4.49	-0.99	-	-	-
P02649	APOE	Apolipoprotein E	3.88	6.67	0.18	-0.49	2.55	2.12
Q13790	APOF	Apolipoprotein F	4.29	9.13	2.72	-	-	-
O95445	APOM	Apolipoprotein M	1.00	3.66	-0.84	-	-	-
P06576	ATP5F1B	ATP synthase subunit beta, mitochondrial	3.87	15.55	-3.08	-	-	-
P25311	AZGP1	Zinc-alpha-2-glycoprotein	-4.53	-0.53	-3.34	-	-	-
P51572	BCAP31	B-cell receptor-associated protein 31	-	-	-	0.31	3.51	7.44
P00736	C1R	Complement C1r subcomponent	1.06	5.40	4.11	-	-	-
P01024	C3	Complement C3	1.73	3.82	0.44	-0.80	2.54	8.29
P0C0L4	C4A	Complement C4-A	2.38	6.59	2.47	2.40	5.27	6.88
P0C0L5	C4B	Complement C4-B	2.32	6.65	3.59	-	-	-
P04003	C4BPA	C4b-binding protein alpha chain	4.33	5.33	4.14	-	-	-
P01031	C5	Complement C5	1.95	4.80	2.99	-	-	-
P07358	C8B	Complement component C8 beta chain	3.06	5.29	0.86	-	-	-
Q8IX12	CCAR1	Cell division cycle and apoptosis regulator protein 1	-	-	-	-6.74	-0.27	-3.63
Q9HCU0	CD248	Endosialin	0.06	5.62	3.32	-	-	-
P60033	CD81	CD81 antigen	3.73	5.79	-0.07	-	-	-
P21926	CD9	CD9 antigen	5.25	9.60	4.43	-	-	-
P08603	CFH	Complement factor H	1.16	-1.70	-5.17	-	-	-
P10909	CLU	Clusterin	1.48	5.77	1.36	-	-	-
Q96KN2	CNDP1	Beta-Ala-His dipeptidase	1.25	3.29	1.60	-	-	-
O75128	COBL	Protein cordon-bleu	-9.71	-14.39	1.20	-	-	-
P39060	COL18A1	Collagen alpha-1(XVIII) chain	7.97	6.74	0.30	-	-	-
Q96IY4	CPB2	Carboxypeptidase B2	7.53	3.38	0.21	-	-	-
P22792	CPN2	Carboxypeptidase N subunit 2	0.53	1.98	3.86	-	-	-
Q9NQ79	CRTAC1	Cartilage acidic protein 1	3.42	9.88	1.07	-	-	-
P35222	CTNNB1	Catenin beta-1	-12.25	-12.52	-12.23	-	-	-
P81605	DCD	Dermcidin	3.75	5.17	-0.87	-	-	-
Q16698	DECR1	2,4-dienoyl-CoA reductase [(3E)-enoyl-CoA-producing], mitochondrial	-	-	-	2.45	-2.40	10.32
O60879	DIAPH2	Protein diaphanous homolog 2	-	-	-	-6.29	-8.93	-7.78

Q16610	ECM1	Extracellular matrix protein 1	-1.86	-11.45	-2.91	-	-	-
P68104	EEF1A1	Elongation factor 1-alpha 1	-	-	-	-32.99	-16.89	1.56
Q05639	EEF1A2	Elongation factor 1-alpha 2	2.69	-0.06	-12.34	-	-	-
P16452	EPB42	Erythrocyte membrane protein band 4.2	-0.63	13.01	0.18	-	-	-
Q96DZ1	ERLEC1	Endoplasmic reticulum lectin 1	-	-	-	2.83	4.60	0.48
P00742	F10	Coagulation factor X	-0.32	3.19	1.92	-	-	-
P00748	F12	Coagulation factor XII	9.42	11.47	9.67	-	-	-
P00488	F13A1	Coagulation factor XIII A chain	1.98	5.26	2.68	-	-	-
Q8N128	FAM177A1	Protein FAM177A1	5.36	14.69	1.64	-	-	-
Q8N0U4	FAM185A	Protein FAM185A	-1.42	-0.07	-8.63	-	-	-
P02675	FGB	Fibrinogen beta chain	0.38	3.67	1.54	-	-	-
P02679	FGG	Fibrinogen gamma chain	1.82	3.34	2.58	-	-	-
P02751	FN1	Fibronectin	0.22	2.38	2.65	-	-	-
Q96EK6	GNPNAT1	Glucosamine 6-phosphate N-acetyltransferase	-2.65	-1.97	-5.46	-	-	-
Q08378	GOLGA3	Golgin subfamily A member 3	-	-	-	6.32	1.14	-0.51
P43304	GPD2	Glycerol-3-phosphate dehydrogenase, mitochondrial	-	-	-	2.61	6.58	8.23
P80108	GPLD1	Phosphatidylinositol-glycan-specific phospholipase D	-0.59	5.32	1.59	-	-	-
P22352	GPX3	Glutathione peroxidase 3	3.69	5.51	4.00	-	-	-
Q14520	HABP2	Hyaluronan-binding protein 2	2.90	5.83	-0.24	-	-	-
P69905	HBA1	Hemoglobin subunit alpha	-0.08	5.93	2.10	-	-	-
P68871	HBB	Hemoglobin subunit beta	0.70	6.71	0.82	-	-	-
P07900	HSP90AA1	Heat shock protein HSP 90-alpha	4.81	6.48	10.72	-	-	-
P35858	IGFALS	Insulin-like growth factor-binding protein complex acid labile subunit	3.37	2.53	-0.16	-	-	-
P06331	IGHV4-34	Immunoglobulin heavy variable 4-34	3.54	3.39	-1.26	-	-	-
P01624	IGKV3-15	Immunoglobulin kappa variable 3-15	4.91	1.84	1.33	-	-	-
B9A064	IGLL5	Immunoglobulin lambda-like polypeptide 5	2.17	5.05	0.21	-	-	-
P06213	INSR	Insulin receptor	8.96	16.89	13.58	-	-	-
P05556	ITGB1	Integrin beta-1	0.36	3.72	4.00	-	-	-
P19827	ITIH1	Inter-alpha-trypsin inhibitor heavy chain H1	1.26	3.64	3.29	-	-	-
P19823	ITIH2	Inter-alpha-trypsin inhibitor heavy chain H2	-0.23	3.95	3.31	-	-	-
Q06033	ITIH3	Inter-alpha-trypsin inhibitor heavy chain H3	0.30	2.43	2.87	-	-	-
Q8IYD2	KLHDC8A	Kelch domain-containing protein 8A	0.50	1.31	7.45	-	-	-
P18428	LBP	Lipopolysaccharide-binding protein	0.33	9.51	-2.87	-	-	-
P04180	LCAT	Phosphatidylcholine-sterol acyltransferase	0.41	4.84	2.47	-	-	-
P02545	LMNA	Prelamin-A/C	-	-	-	0.26	0.24	0.82
P08519	LPA	Apolipoprotein(a)	6.26	4.42	4.32	3.66	4.51	2.05
P02750	LRG1	Leucine-rich alpha-2-glycoprotein	-7.23	-6.42	0.34	-	-	-

O75581	LRP6	Low-density lipoprotein receptor-related protein 6	-9.38	-7.69	-3.20	-	-	-
Q13477	MADCAM1	Mucosal addressin cell adhesion molecule 1	4.03	6.52	0.49	-	-	-
P42679	MATK	Megakaryocyte-associated tyrosine-protein kinase	-	-	-	5.86	8.14	7.01
P49736	MCM2	DNA replication licensing factor MCM2	-	-	-	6.25	5.38	5.57
P40925	MDH1	Malate dehydrogenase, cytoplasmic	-10.30	-0.75	-7.47	-	-	-
Q9BUN1	MENT	Protein MENT	2.68	7.84	1.38	-	-	-
Q8IWI9	MGA	MAX gene-associated protein	8.57	7.83	-3.89	-	-	-
Q14149	MORC3	MORC family CW-type zinc finger protein 3	8.06	5.00	7.60	-	-	-
Q9Y2A7	NCKAP1	Nck-associated protein 1	-	-	-	10.64	5.48	10.58
P13591	NCAM1	Neural cell adhesion molecule 1	0.12	7.33	2.36	-	-	-
P19338	NCL	Nucleolin	-	-	-	-1.30	0.98	2.06
O00541	PES1	Pescadillo homolog	-	-	-	-5.05	-1.06	0.28
P00558	PGK1	Phosphoglycerate kinase 1	-	-	-	-4.61	-1.17	-7.30
Q96PD5	PGLYRP2	N-acetylmuramoyl-L-alanine amidase	-2.16	0.42	-2.03	-	-	-
Q8WV24	PHLDA1	Pleckstrin homology-like domain family A member 1	-3.65	-10.42	-2.26	-	-	-
Q13093	PLA2G7	Platelet-activating factor acetylhydrolase	3.52	3.33	1.68	-	-	-
P55058	PLTP	Phospholipid transfer protein	1.21	2.82	1.26	-	-	-
O00592	PODXL	Podocalyxin	10.48	15.07	12.23	-	-	-
P27169	PON1	Serum paraoxonase/arylesterase 1	3.81	9.82	3.84	6.57	8.83	7.68
Q15166	PON3	Serum paraoxonase/lactonase 3	0.05	3.91	1.98	-	-	-
P14314	PRKCSH	Glucosidase 2 subunit beta	2.13	12.94	10.59	-	-	-
P07225	PROS1	Vitamin K-dependent protein S	1.09	5.93	2.25	-	-	-
P22891	PROZ	Vitamin K-dependent protein Z	-0.03	8.70	3.59	-	-	-
P07602	PSAP	Prosaposin	4.34	9.37	4.13	-	-	-
P51665	PSMD7	26S proteasome non-ATPase regulatory subunit 7	-	-	-	2.31	13.27	0.40
P49792	RANBP2	E3 SUMO-protein ligase RanBP2	-	-	-	6.48	11.15	15.96
P02753	RBP4	Retinol-binding protein 4	2.57	3.66	1.56	-	-	-
Q96DB5	RMDN1	Regulator of microtubule dynamics protein 1	-	-	-	5.51	6.04	3.52
P0DJI9	SAA2	Serum amyloid A-2 protein	0.04	4.85	1.95	-	-	-
P35542	SAA4	Serum amyloid A-4 protein	-	-	-	-5.53	-2.61	-2.37
O00560	SDCBP	Syntenin-1	1.34	7.33	7.43	-	-	-
P01009	SERPINA1	Alpha-1-antitrypsin	2.23	4.81	0.23	-1.08	3.18	3.22
Q9UK55	SERPINA10	Protein Z-dependent protease inhibitor	2.64	6.02	1.88	-	-	-
P20848	SERPINA2	Putative alpha-1-antitrypsin-related protein	6.08	7.27	12.96	-	-	-
P29622	SERPINA4	Kallistatin	-3.22	-4.49	-1.83	-	-	-
P08185	SERPINA6	Corticosteroid-binding globulin	-5.30	-1.54	-10.97	-	-	-
P08697	SERPINF2	Alpha-2-antiplasmin	1.13	4.07	-1.05	-	-	-

P05155	SERPING1	Plasma protease C1 inhibitor	0.56	6.08	3.91	-	-	-
P02730	SLC4A1	Band 3 anion transport protein	-0.12	11.08	4.80	-	-	-
Q8IY18	SMC5	Structural maintenance of chromosomes protein 5	-	-	-	-7.74	-3.01	-2.68
Q13573	SNW1	SNW domain-containing protein 1	-	-	-	-1.61	1.27	0.65
Q14515	SPARCL1	SPARC-like protein 1	1.29	4.67	-2.36	-	-	-
Q13103	SPP2	Secreted phosphoprotein 24	7.90	8.37	8.61	-	-	-
Q9UQ35	SRRM2	Serine/arginine repetitive matrix protein 2	-	-	-	0.36	1.42	4.35
P61764	STXBP1	Syntaxin-binding protein 1	-	-	-	-1.63	2.20	6.45
P02786	TFRC	Transferrin receptor protein 1	0.53	3.83	6.59	-	-	-
Q9H3N1	TMX1	Thioredoxin-related transmembrane protein 1	-	-	-	-17.84	-9.83	-18.23
P09493	TPM1	Tropomyosin alpha-1 chain	-1.47	5.77	5.10	-	-	-
P06753	TPM3	Tropomyosin alpha-3 chain	1.35	14.45	0.61	-	-	-
P67936	TPM4	Tropomyosin alpha-4 chain	0.55	6.31	2.15	-	-	-
P02766	TTR	Transthyretin	3.22	2.92	3.00	-	-	-
P55089	UCN	Urocortin	1.73	3.74	1.06	-	-	-
Q9Y5T5	USP16	Ubiquitin carboxyl-terminal hydrolase 16	-	-	-	5.94	5.43	5.52
Q9ULK5	VANGL2	Vang-like protein 2	-	-	-	1.13	1.45	-0.99
P04004	VTN	Vitronectin	0.31	3.79	2.29	-	-	-
P63104	YWHAZ	14-3-3 protein zeta/delta	-1.63	3.97	4.40	-	-	-
P0DOX6	N/A	Immunoglobulin mu heavy chain	5.43	5.72	2.40	-	-	-

Supplemental table I. List of all proteins identified and relatively quantified.
The fold differences were calculated as ratio of the peak intensities of a protein in Lp(a) over that in LDL and are presented as log2 of the fold differences. A log2 value bigger than 1 means the protein was at least 2 fold more abundant in Lp(a) than in LDL of the same donor, a value smaller than -1 means the protein was at least 2-fold more abundant in LDL than in Lp(a) derived from the same donor. Lipoproteins were isolated from the same three donors twice and compared between LDL and Lp(a) of the same isolation.

	pmol/ug protein					
	Donor / Particle					
	A		B		C	
**Lipid Class**	**LDL**	**Lp(a)**	**LDL**	**Lp(a)**	**LDL**	**Lp(a)**
**CER**	1.5	4.2	2.9	3.9	15.4	2.5
**DAG**	22.5	13.9	6.9	59.1	7.5	6.9
**LPC**	3.8	1.9	4.3	3.4	6.7	5.1
**PC**	810.7	461.4	654.5	496.9	731.3	432.4
**PC O-**	22.4	12.7	28.9	22.1	43.0	24.3
**PC Ox**	16.9	32.2	6.5	25.9	0.7	28.0
**SE**	1 047.7	1 284.5	1 563.8	1 492.6	2 576.6	1 443.4
**SM**	140.2	171.2	157.4	159.5	279.2	207.7
**TAG**	103.9	201.0	122.9	218.4	120.9	64.9

Supplemental table II. Lipid class profiles of the particles per donor.
Lipid classes were analysed by LC-MS and are presented as pmol/µg protein.

				Values as pmol/µg Donor / Particle					
				A		B		C	
Lipid class	Lipid species	m/z	RT	LDL	Lp(a)	LDL	Lp(a)	LDL	Lp(a)
CER	CER 16:0	538.5	6.9	0.11	0.13	0.11	0.13	1.31	0.15
	CER 18:0	566.5	7.0	0.02	0.05	0.02	0.09	0.25	0.11
	CER 18:1	564.5	7.4	0.02	0.03	0.02	0.03	0.02	0.01
	CER 20:0	594.5	7.9	0.03	0.07	0.08	0.07	0.36	0.09
	CER 20:1	592.5	8.3	0.01	0.02	0.03	0.01	0.01	0.02
	CER 22:0	622.6	8.9	0.23	0.47	0.39	0.53	2.94	0.35
	CER 22:1	620.5	9.0	0.01	0.01	0.01	0.00	0.01	0.00
	CER 24:0	650.6	10.5	0.68	2.53	1.61	2.28	7.48	0.67
	CER 24:1	648.6	9.7	0.39	0.86	0.58	0.77	3.03	1.07
DAG	DAG 32:1	582.5	8.2	1.49	0.61	0.76	4.41	0.57	0.48
	DAG 32:2	584.5	6.1	1.60	0.53	0.72	4.92	0.64	0.32
	DAG 34:1	612.5	8.5	2.01	0.97	0.59	4.97	0.36	0.49
	DAG 34:2	610.5	6.9	4.85	1.90	1.02	9.53	0.97	0.87
	DAG 36:1	640.5	8.6	1.28	1.11	0.71	4.14	0.37	0.57
	DAG 36:2	638.5	7.8	8.22	7.44	1.71	21.53	3.30	2.92
	DAG 38:4	660.5	7.0	1.29	0.59	0.67	5.61	0.85	0.91
	DAG 38:5	662.5	6.7	1.77	0.81	0.72	3.98	0.43	0.38
LPC	LPC 14:0	468.5	0.5	0.00	0.00	0.00	0.17	0.05	0.14
	LPC 16:0	496.5	1.5	1.48	0.61	2.53	1.81	3.57	3.23
	LPC 16:1	494.5	0.4	0.00	0.00	0.00	0.28	0.11	0.00
	LPC 18:0	524.5	2.3	1.43	0.66	0.42	0.75	1.43	0.96
	LPC 18:1	522.5	1.6	0.93	0.65	1.13	0.34	1.03	0.47
	LPC 18:2	520.5	0.6	0.00	0.00	0.19	0.00	0.50	0.27
PC	PC 32:0	734.7	7.0	4.04	4.54	4.53	4.64	6.84	5.41
	PC 32:1	732.6	6.4	11.61	8.36	3.57	3.26	7.96	5.72
	PC 32:2	730.6	6.1	0.75	0.74	0.58	0.68	1.40	0.94
	PC 34:0	762.7	7.7	0.63	0.91	1.03	1.21	1.34	1.38
	PC 34:1	760.7	7.0	134.17	125.27	88.77	87.97	104.80	75.98
	PC 34:2	758.7	6.5	146.25	98.16	149.32	134.36	175.36	117.29
	PC 34:3	756.7	6.0	9.99	6.00	6.28	5.55	10.81	6.82
	PC 36:1	788.7	8.4	5.10	5.93	4.87	4.53	11.92	8.97
	PC 36:2	786.7	7.1	89.59	54.71	88.03	76.99	119.91	72.29
	PC 36:3	784.7	6.6	76.75	38.91	50.50	42.57	67.59	39.92
	PC 36:4	782.7	6.3	119.71	35.88	91.17	33.17	64.33	28.87
	PC 36:5	780.7	5.9	15.37	3.84	27.22	10.08	10.55	3.26
	PC 36:6	778.7	5.5	0.14	0.07	0.27	0.08	0.16	0.11
	PC 38:2	814.7	8.4	11.23	12.12	8.41	8.26	15.21	10.44
	PC 38:3	812.7	7.3	23.58	10.33	12.85	10.29	22.53	12.42
	PC 38:4	810.7	7.0	68.36	24.88	31.08	19.74	45.84	19.53
	PC 38:5	808.7	6.4	33.71	10.51	29.95	18.51	24.75	9.44
	PC 38:6	806.7	6.1	38.79	13.84	40.47	26.52	26.10	8.59
	PC 38:7	804.7	5.5	0.23	0.09	0.51	0.31	0.35	0.12
	PC 40:1	844.7	7.5	0.03	0.04	0.08	0.02	0.03	0.08
	PC 40:2	842.7	9.1	0.06	0.16	0.09	0.06	0.04	0.12
	PC 40:3	840.7	8.0	0.08	0.11	0.04	0.10	0.11	0.03
	PC 40:4	838.7	7.4	1.72	0.87	0.68	0.42	1.71	0.77
	PC 40:5	836.7	7.0	5.36	1.99	4.34	2.54	3.31	1.42
	PC 40:6	834.7	6.8	9.01	2.12	6.82	3.05	4.89	1.32

	PC 40:7	832.7	6.1	3.15	0.76	2.43	1.55	2.27	0.51
	PC 40:8	830.7	5.8	0.31	0.10	0.19	0.05	0.28	0.22
	PC 44:12	878.8	8.8	1.01	0.19	0.46	0.35	0.95	0.45
	PC-O 32:0	720.6	7.5	0.45	0.53	0.60	0.59	1.01	0.87
	PC-O 32:1	718.6	7.4	0.22	0.28	0.55	0.45	0.81	0.47
	PC-O 34:0	748.7	8.1	0.25	0.07	0.17	0.13	0.17	0.20
	PC-O 34:1	746.7	7.5	1.92	1.42	1.52	1.61	3.03	2.26
	PC-O 34:2	744.7	6.9	0.87	0.61	2.32	2.24	2.67	1.56
	PC-O 34:3	742.7	6.8	1.12	0.72	2.48	1.91	2.88	1.51
	PC-O 36:1	774.7	8.4	0.04	0.05	1.06	0.88	0.58	1.53
	PC-O 36:2	772.7	7.6	0.47	0.64	0.90	0.73	1.50	0.98
	PC-O 36:3	770.7	7.0	0.71	0.32	2.19	1.74	2.68	1.64
	PC-O 36:4	768.7	6.7	3.28	1.75	3.75	2.99	6.57	2.85
	PC-O 36:5	766.7	6.6	2.73	1.46	3.78	2.22	4.13	1.97
	PC-O 36:6	764.7	6.4	0.24	0.21	0.87	0.49	0.35	0.16
	PC-O 38:2	800.7	8.0	1.03	1.21	1.26	1.11	2.64	2.26
	PC-O 38:3	798.7	7.8	0.18	0.13	0.14	0.04	0.18	0.29
	PC-O 38:4	796.7	7.4	1.62	0.76	0.93	0.69	4.32	1.42
	PC-O 38:5	794.7	6.7	3.64	0.87	2.72	1.88	4.79	1.99
	PC-O 38:6	792.7	6.6	1.62	0.37	1.97	1.01	1.71	0.54
	PC-O 40:3	826.7	8.2	0.10	0.09	0.27	0.07	0.39	0.27
	PC-O 40:4	824.7	8.1	0.18	0.15	0.16	0.16	0.62	0.45
	PC-O 40:5	822.7	7.4	0.62	0.13	0.45	0.29	0.94	0.26
	PC-O 40:6	820.7	7.2	0.34	0.27	0.20	0.07	0.53	0.13
	PC-O 40:7	818.7	7.0	0.77	0.63	0.60	0.73	0.46	0.71
PC(ox)	PC 21:1+O	594.3	1.0	0.07	0.17	0.05	0.10	0.01	0.05
	PC 21:1+O2	610.5	1.6	0.01	0.04	0.01	0.03	0.00	0.02
	PC 22:1+O	608.4	1.5	0.02	0.01	0.01	0.04	0.00	0.01
	PC 23:1+O	622.5	1.6	0.11	0.03	0.00	0.05	0.00	0.06
	PC 24:2+O2	650.6	1.9	0.35	0.00	0.02	0.35	0.03	0.06
	PC 24:2+O3	666.4	1.8	0.04	0.12	0.03	0.05	0.01	0.08
	PC 24:3+O2	648.5	1.6	0.02	0.02	0.00	0.02	0.01	0.02
	PC 24:3+O3	664.4	1.6	0.08	0.03	0.00	0.04	0.00	0.10
	PC 26:2+O2	678.5	4.6	0.07	0.01	0.04	0.13	0.00	0.15
	PC 26:3+O2	676.5	2.4	0.07	0.00	0.00	0.04	0.00	0.05
	PC 34:2+O	774.6	4.5	1.62	2.05	0.71	2.53	0.04	2.68
	PC 34:2+O2	790.2	4.8	4.37	8.49	0.37	8.51	0.08	10.72
	PC 36:2+O	802.5	7.0	0.78	0.83	0.81	1.21	0.03	1.37
	PC 36:3+O3	830.5	3.5	0.07	0.11	0.05	0.10	0.01	0.28
	PC 36:4+O	798.6	5.3	2.05	3.68	1.62	2.37	0.05	2.39
	PC 36:4+O2	810.5	4.8	1.75	5.91	0.51	2.69	0.01	2.30
	PC 36:4+O3	828.5	3.5	0.06	0.50	0.12	0.51	0.02	0.99
	PC 36:4+O4	842.6	3.0	0.18	0.38	0.08	0.25	0.00	0.57
	PC 36:5+O3	826.6	2.9	0.49	0.40	0.08	0.24	0.01	0.33
	PC 38:4+O	814.6	5.6	1.03	1.97	1.08	1.33	0.03	1.59
	PC 38:4+O2	840.6	5.8	1.06	3.20	0.27	1.70	0.34	2.04
	PC 38:4+O3	846.6	4.4	0.40	0.54	0.02	0.23	0.00	0.16
	PC 38:4+O4	858.6	4.2	0.26	0.38	0.01	0.16	0.00	0.20
	PC 38:5+O2	838.6	4.7	0.95	1.89	0.37	1.56	0.00	1.11
	PC 38:6+O2	832.5	4.8	1.03	1.39	0.23	1.62	0.01	0.68

CE	CE 16:0	642.6	13.4	53.82	83.63	84.49	86.09	119.03	79.81
	CE 16:1	640.6	12.6	21.81	43.15	15.31	12.38	59.55	45.90
	CE 17:0	656.6	14.2	0.48	0.00	0.00	1.63	3.94	1.62
	CE 17:1	654.6	12.9	0.82	2.54	0.99	0.95	6.25	4.97
	CE 18:0	670.6	14.2	0.14	0.67	1.89	0.63	5.44	2.94
	CE 18:1	668.6	13.4	313.96	399.80	272.98	285.90	445.85	304.36
	CE 18:2	666.6	12.7	542.02	667.92	1 055.66	1 047.89	1 580.03	912.59
	CE 18:3	664.6	12.1	7.65	6.59	8.31	1.09	63.91	14.45
	CE 20:1	696.6	13.9	0.00	4.82	2.45	3.83	1.52	0.00
	CE 20:2	694.6	13.4	0.59	0.86	2.08	1.39	1.61	1.12
	CE 20:3	692.6	12.6	3.17	11.84	15.17	8.87	28.85	11.92
	CE 20:4	690.6	12.3	99.04	58.45	92.24	35.31	244.62	59.41
	CE 20:5	688.6	11.7	1.62	2.23	9.31	3.35	9.25	1.27
	CE 22:4	718.6	8.2	0.00	0.65	0.03	0.00	0.55	0.51
	CE 22:5	716.6	10.0	0.54	0.25	0.00	1.08	1.34	1.15
	CE 22:6	714.6	11.9	2.06	1.14	2.90	2.21	4.88	1.34
SM	SM 14:0	675.7	5.5	0.94	2.46	2.03	2.93	4.98	2.67
	SM 14:1	673.6	4.0	0.19	0.10	0.11	0.04	0.23	0.09
	SM 15:0	689.7	5.9	1.33	2.23	2.58	3.28	4.27	2.96
	SM 15:1	687.7	6.1	0.06	0.04	0.03	0.04	0.04	0.09
	SM 16:0	703.7	6.2	21.01	32.97	30.00	32.71	49.08	39.63
	SM 16:0dh	705.7	6.8	0.03	0.18	1.94	0.05	1.76	1.74
	SM 16:1	701.7	5.6	2.71	3.74	2.05	3.75	6.36	3.53
	SM 17:0	717.7	6.6	0.58	1.05	1.11	0.90	1.53	1.50
	SM 17:0dh	727.7	7.9	0.15	0.07	0.10	0.20	0.53	0.31
	SM 17:1	715.7	6.0	0.24	0.14	0.15	0.23	0.41	0.29
	SM 18:0	733.7	7.3	0.63	0.03	0.66	0.14	1.69	1.17
	SM 18:0dh	757.7	8.0	1.53	2.00	2.28	2.37	3.44	2.57
	SM 18:1	731.7	7.0	5.79	6.83	7.62	7.33	10.46	7.13
	SM 18:2	729.7	6.3	2.24	2.77	2.57	3.13	4.50	2.99
	SM 19:0	759.7	7.7	5.01	7.03	6.64	6.76	13.15	8.51
	SM 20:0	785.7	7.7	7.97	8.79	10.69	8.29	18.86	11.60
	SM 20:1	773.7	8.0	1.72	2.27	2.62	2.93	6.05	5.02
	SM 21:0	787.7	8.4	17.63	18.59	18.89	15.77	37.84	27.88
	SM 22:0	801.5	8.8	5.62	8.34	5.54	8.36	11.93	13.77
	SM 22:1	799.7	8.0	4.19	4.54	5.09	4.47	9.44	6.88
	SM 23:0	813.7	8.4	38.60	38.20	31.52	31.19	50.13	30.00
	SM 23:1	811.7	7.7	9.25	9.42	9.36	9.59	17.09	10.11
	SM 24:0	829.7	9.5	0.71	1.66	0.91	1.02	1.18	1.74
	SM 24:1	827.7	8.7	1.66	0.06	1.21	1.24	2.02	1.30
	SM 24:2	815.7	9.2	10.18	17.31	11.26	12.32	21.81	23.60
	SM 26:0	843.7	10.0	0.05	0.09	0.05	0.23	0.08	0.24
	SM 26:1	841.7	9.1	0.15	0.32	0.36	0.25	0.34	0.31
TAG	TAG 48:0	824.7	13.2	0.19	0.80	0.81	1.19	0.51	0.59
	TAG 48:1	822.7	12.5	0.67	2.07	2.18	3.91	2.33	1.76
	TAG 48:2	820.7	11.9	0.68	1.96	1.76	2.97	1.99	1.07
	TAG 48:3	818.7	11.6	0.10	0.39	0.09	0.22	0.36	0.15
	TAG 50:0	852.7	13.9	0.00	0.00	0.13	0.00	0.50	0.25
	TAG 50:1	848.7	13.2	3.06	9.79	5.84	11.29	5.37	4.26
	TAG 50:2	846.7	12.5	7.12	14.12	10.69	14.52	8.91	6.55

TAG 50:3	844.7	11.9	2.31	3.94	4.37	3.67	3.97	1.97
TAG 50:4	842.7	11.5	0.04	0.47	0.33	0.96	0.67	0.27
TAG 52:1	878.7	13.8	0.86	5.09	1.75	2.81	2.43	1.57
TAG 52:2	876.7	13.1	37.46	73.62	38.06	71.33	33.21	20.76
TAG 52:3	874.7	12.6	13.17	31.66	18.71	35.91	17.46	9.09
TAG 52:4	872.7	12.3	1.90	1.62	1.93	11.06	7.28	2.53
TAG 52:5	870.7	9.7	0.30	0.86	0.36	0.00	1.25	0.53
TAG 52:6	868.7	3.7	0.00	0.00	0.00	0.00	0.20	0.09
TAG 54:1	906.7	9.6	0.12	0.00	0.21	0.00	0.11	0.24
TAG 54:2	904.7	13.7	2.51	6.69	1.76	3.78	3.93	1.92
TAG 54:3	902.7	13.1	5.95	10.45	6.58	9.83	3.98	2.09
TAG 54:4	900.7	12.6	6.00	11.40	6.54	12.27	7.73	2.88
TAG 54:5	898.7	12.1	4.61	5.37	4.55	8.29	5.11	1.43
TAG 54:6	896.7	11.8	1.26	0.76	0.83	2.29	1.07	0.26
TAG 54:7	894.7	11.2	0.14	0.24	0.11	0.79	0.15	0.06
TAG 56:3	930.7	13.7	0.40	0.50	0.15	0.16	0.53	0.20
TAG 56:4	928.7	13.2	1.11	0.85	0.96	0.84	1.00	0.00
TAG 56:5	926.7	13.0	1.56	2.11	0.95	1.92	2.04	0.63
TAG 56:6	924.7	12.3	2.65	2.54	2.10	2.99	2.27	0.39
TAG 56:7	922.7	11.9	1.16	1.01	1.45	1.42	1.07	0.18
TAG 56:8	920.7	9.5	0.42	0.68	0.20	0.60	0.00	0.11
TAG 56:9	946.7	9.3	1.52	3.36	1.54	2.94	1.26	0.84
TAG 56:10	944.7	9.1	2.75	4.29	2.93	3.96	1.35	0.64
TAG 56:11	942.7	9.0	1.66	2.06	2.05	2.97	1.08	0.83
TAG 56:12	940.7	8.5	1.38	1.40	2.49	2.67	1.15	0.53
TAG 56:13	938.6	8.0	0.56	0.54	0.50	0.85	0.47	0.04

Supplemental table III. Lipid species of the particles per donor.
Lipids were analysed by LC-MS and are presented as pmol/µg protein.
